# Diurnal variations of resting-state fMRI data: A graph-based analysis

**DOI:** 10.1016/j.neuroimage.2022.119246

**Published:** 2022-04-25

**Authors:** Farzad V. Farahani, Waldemar Karwowski, Mark D’Esposito, Richard F. Betzel, Pamela K. Douglas, Anna Maria Sobczak, Bartosz Bohaterewicz, Tadeusz Marek, Magdalena Fafrowicz

**Affiliations:** a Department of Biostatistics, Johns Hopkins University, Baltimore, MD, USA; b Computational Neuroergonomics Laboratory, Department of Industrial Engineering and Management Systems, University of Central Florida, Orlando, FL, USA; c Helen Wills Neuroscience Institute, University of California, Berkeley, CA, USA; d Department of Psychology, University of California, Berkeley, CA, USA; e Department of Psychological and Brain Sciences, Indiana University, Bloomington, IN, USA; f Institute for Simulation and Training, University of Central Florida, Orlando, FL, USA; g Department of Psychiatry and Biobehavioral Sciences, University of California, Los Angeles, Los Angeles, CA, USA; h Department of Cognitive Neuroscience and Neuroergonomics, Institute of Applied Psychology, Jagiellonian University, Krakow, Poland; i Department of Psychology of Individual Differences, Psychological Diagnosis, and Psychometrics, Institute of Psychology, University of Social Sciences and Humanities, Warsaw, Poland; j Malopolska Centre of Biotechnology, Jagiellonian University, Krakow, Poland

**Keywords:** Functional connectivity, Resting-state fMRI, Graph theory, Network analysis, Circadian rhythm, Chronotype, Brain networks

## Abstract

Circadian rhythms (lasting approximately 24 h) control and entrain various physiological processes, ranging from neural activity and hormone secretion to sleep cycles and eating habits. Several studies have shown that time of day (TOD) is associated with human cognition and brain functions. In this study, utilizing a chronotype-based paradigm, we applied a graph theory approach on resting-state functional MRI (rs-fMRI) data to compare whole-brain functional network topology between morning and evening sessions and between morning-type (MT) and evening-type (ET) participants. Sixty-two individuals (31 MT and 31 ET) underwent two fMRI sessions, approximately 1 hour (morning) and 10 h (evening) after their wake-up time, according to their declared habitual sleep-wake pattern on a regular working day. In the global analysis, the findings revealed the effect of TOD on functional connectivity (FC) patterns, including increased small-worldness, assortativity, and synchronization across the day. However, we identified no significant differences based on chronotype categories. The study of the modular structure of the brain at mesoscale showed that functional networks tended to be more integrated with one another in the evening session than in the morning session. Local/regional changes were affected by both factors (i.e., TOD and chronotype), mostly in areas associated with somatomotor, attention, frontoparietal, and default networks. Furthermore, connectivity and hub analyses revealed that the somatomotor, ventral attention, and visual networks covered the most highly connected areas in the morning and evening sessions: the latter two were more active in the morning sessions, and the first was identified as being more active in the evening. Finally, we performed a correlation analysis to determine whether global and nodal measures were associated with subjective assessments across participants. Collectively, these findings contribute to an increased understanding of diurnal fluctuations in resting brain activity and highlight the role of TOD in future studies on brain function and the design of fMRI experiments.

## 1. Introduction

Circadian rhythms are endogenous oscillations with a periodicity of approximately 24 h in most living organisms. They have an impact on a variety of physiological phenomena, including the sleep-wake cycle (Borbély, 1982; Dijk and Lockley, 2002; Schmidt et al., 2012), body temperature (Refinetti and Menaker, 1992), endocrine and metabolic rhythms (Hastings et al., 2007), gene expression (Storch et al., 2002), and musculoskeletal activity (Aoyama and Shibata, 2017), as well as a wide range of brain functions (Dibner et al., 2010; Schmidt et al., 2007). Studies of brain function in humans have shown that circadian variations also have an impact on a wide variety of abilities, such as attention (Valdez et al., 2005), working memory (Ramírez et al., 2006), motor (Edwards et al., 2007), and visual detection (Tassi et al., 2000). These studies investigated function using multiple scales of brain organization, from the level of individual cells and synapses (Gilestro et al., 2009; Kuhn et al., 2016; Vyazovskiy et al., 2008) to brain regions and large-scale functional connectivity (Blautzik et al., 2013; Hodkinson et al., 2014; Orban et al., 2020; Shannon et al., 2013; Steel et al., 2019).

The chronotype-based paradigm is susceptible to circadian and homeostatic rhythms, which provides a suitable way to measure the effects of sleep-wake regulation on cerebral mechanisms (Schmidt et al., 2007). A chronotype—a biologically driven circadian typology—refers to individual differences in sleep-wake cycles, diurnal preferences, and alertness throughout the day (Roenneberg et al., 2003; Susman et al., 2007). Questionnaires have reliably confirmed the differences in chronotypes (Adan and Almirall, 1991; Horne and Östberg, 1976), which have been shown to be strongly correlated with the physiological properties of circadian rhythms, including melatonin levels, core body temperature, rest/activity cycles, midsleep point, heart rate, blood pressure, and physical activity (Adan et al., 2012; Anderson et al., 2017; Facer-Childs and Brandstaetter, 2015; Roeser et al., 2012). Genetic factors have been shown to associate with diurnal preference and homeostatic regulation of sleep. One of the most extensively studied clock genes is the primate-specific gene PERIOD3 (PER3). In humans, the variable number tandem repeat (VNTR) in the PER3 gene, consisting of either 4 or 5 repeated 54 base pair sequences encoding 18 amino acids, affects circadian typology and sleep homeostatic drive (for a review, see Archer et al., 2018).

Traditionally, individuals fall into the morning (“early larks”) or evening (“night owls”) chronotypes. Morning chronotypes typically have physiological and mental peaks that are shifted toward the earlier hours of the day, while evening chronotypes are more active later in the day (Bailey and Heitkemper, 2001; Kerkhof and Dongen, 1996). There is some evidence that polymorphism of the PER3 gene is associated with individual differences in circadian and sleep phenotypes. The PER3 4-repeat allele has been associated with “eveningness,” whereas PER3 5/5 is linked with “morningness” and greater homeostatic sleep pressure (e.g., Archer et al., 2018; Liberman et al., 2017; Viola et al., 2007). Various studies have shown that people with different chronotypes have significantly different diurnal profiles of cognition and behavior (Horne et al., 1980; Norbury, 2020; Schmidt et al., 2007; Valdez et al., 2012). Circadian variations in performance-related neural activity have been reported in studies utilizing chronotype-based paradigms (e.g., Facer-Childs et al., 2019b; Fafrowicz et al., 2009; Gorfine et al., 2007; Peres et al., 2011; Schmidt et al., 2009, 2012, 2015; Vandewalle et al., 2009, 2011).

Although extensive research has been carried out on the effects of circadian rhythms on behavior, few studies have investigated the impact of time of day (TOD) and chronotype on functional magnetic resonance imaging (fMRI) activity (Blautzik et al., 2013; Cordani et al., 2018; Fafrowicz et al., 2019; Gorfine and Zisapel, 2009; Hodkinson et al., 2014; Jiang et al., 2016; Marek et al., 2010; Peres et al., 2011; Schmidt et al., 2012; Shannon et al., 2013; Steel et al., 2019). The studies that have been performed often yielded contradictory or even ambiguous findings. Also, most fMRI studies assume that diurnal fluctuations of brain connectivity patterns and human chronotypes are relatively in-significant and unlikely to lead to a substantial systematic bias when performing group analyses (Orban et al., 2020).

Here, we tried to bridge the gap using a graph-based approach. In recent years, the application of graph theory in neuroimaging studies to analyze the human brain connectome has received much attention (Bassett and Bullmore, 2009; Bullmore and Sporns, 2012, 2009; Farahani et al., 2019b; Rubinov and Sporns, 2010). In terms of circadian rhythms, the few studies that have been conducted using graph theory (Anderson et al., 2017; Farahani et al., 2019a) mainly focused on task-based data and did not consider chronotypes. However, many other cognitive and behavioral studies have performed analyses of network properties that feature graph theory, including those focused on human intelligence (Hilger et al., 2017), lifetime trajectory (Finotelli et al., 2018; Gozdas et al., 2018), working memory performance (Markett et al., 2018), perception (Sadaghiani et al., 2015), and fatigue (Petruo et al., 2018). Graph theory has also been applied to the study of a wide range of neurological and psychiatric disorders, including epilepsy (Evangselisti et al., 2018), Alzheimer’s disease (Hojjati et al., 2017), multiple sclerosis (Eijlers et al., 2017), autism (Sadeghi et al., 2017), and attention-deficit/hyperactivity disorder (dos Santos Siqueira et al., 2014).

This paper is an extension of the work published by Farahani et al. (2021), and it utilizes the same experimental dataset, though it introduces significant methodological improvements. Here, using concepts from network neuroscience, we examined the effect of TOD on resting-state fMRI (rs-fMRI) functional connectivity while taking into account subject chronotypes. We compared the brain network properties—at different topological scales including local, meso and global—between the morning and evening sessions, as well as “early larks” and “night owls.” We found greater small-worldness, assortativity, and synchronization as the waking time increased, although there was no chronotype effect. In the mesoscale analysis, we found that systems were more inclined to integrate in the evening than in the morning. The local/regional analysis revealed significant changes in both factors under study—TOD and chronotype—which were primarily associated with the somatomotor, attention, control, and default-mode networks. Furthermore, due to the homogeneity inefficiency in the parcellation we used previously (i.e., the automated anatomical labeling atlas; Tzourio-Mazoyer et al., 2002)—which may not represent the structure of resting-state functional connectivity (FC) well (Craddock et al., 2012; Gordon et al., 2016; Shen et al., 2013)—in this study, we identified areas of interest using cortical Schaefer/Yeo parcellation (Schaefer et al., 2018), in which each node is preassigned to a functional network. As a result, we noticed significant changes, particularly in the local and mesoscale analyses, that were not detected in the previous study. Within the context of our findings, it is clear that TOD may influence connectivity patterns in resting-state fMRI data, making this variable a potentially vital factor to consider in future rs-fMRI experiments.

## 2. Methods

### 2.1. Participants and study procedures

Participants were recruited through online advertisements on the laboratory’s website and Facebook page. A total of 5354 volunteers participated in the first stage of selection; they were asked to complete three questionnaires: the Chronotype Questionnaire (Oginska et al., 2017) for assessing diurnal preferences, the Epworth Sleepiness Scale (ESS; Johns, 1991) for measuring daytime sleepiness, and the sleep-wake assessment (real versus ideal wake-up and bedtimes). Individuals reporting excessive daytime sleepiness were excluded from the study, as determined by a cutoff ESS score of 10 or fewer points. Four hundred fifty-one participants were divided into morning or evening chronotypes. All participants underwent genotyping to identify *PER3* VNTR polymorphisms in DNA isolated from buccal swabs using a DNA Gen-eMATRIX Swab-Extract DNA Purification Kit (EURx, Gdańsk, Poland) according to the manufacturer’s protocol. Only individuals who were homozygous for the *PER3 4* (evening-type [ET] circadian typology) and *PER3 5* (morning-type [MT] circadian typology) alleles were included in the study. Other selection criteria included an age between 20 and 35 years, right-handedness—as indicated by the Edinburgh Handedness Inventory (Oldfield, 1971)—a regular TOD schedule with no sleep debt, no neurological or psychiatric disorders, no addiction, normal or corrected-to-normal vision, and no contraindications to magnetic resonance imaging (MRI). We identified 73 young, healthy participants (39 women; age: 23.97 ± 3.26 years) who met these criteria and selected them for the study. Demographic information and the results of the questionnaires are provided in Table 1.

Resting-state fMRI was performed twice, in a morning and evening session, about 1 and 10 h, respectively, after awakening from nighttime sleep. The order of the sessions was counterbalanced across the study sample. The participants were asked to maintain a regular sleep-wake schedule for one week before the study, which was monitored using Motion Watch 8 actigraphs. These actigraphs were also worn to supervise participants’ sleep length and quality during the study days. Actigraphy results are provided in Table 2. The night before the morning sessions, the participants slept in the same building as the MRI scanner. All participants abstained from alcohol (48 h) and caffeine (24 h) before the MRI scanning sessions and were only allowed to engage in non-strenuous activities during study days. The study was approved by the Institute of Applied Psychology Ethics Committee of the Jagiellonian University (Krakow, Poland). Written informed consent was provided by all participants in accordance with the Declaration of Helsinki.

### 2.2. Data acquisition

MRI studies were conducted using a 3T Siemens Skyra MR system equipped with a 64-channel head coil. Anatomical data were acquired through a sagittal 3-dimensional T1-weighted MPRAGE sequence. Ten minutes of resting-state blood oxygenation level-dependent (BOLD) images were scanned using a gradient-echo single-shot echo-planar imaging sequence with the following parameters: repetition time (TR) = 1800 ms; echo time (TE) = 27 ms; field of view (FOV) = 256 × 256 mm^2^; slice thickness = 4 mm; and voxel size = 4 × 4 × 4 mm^3^ with no gap. A total of 34 interleaved transverse slices and 335 vol were collected for each participant. The subjects were instructed to remain awake with their eyes open and to avoid thinking of anything deliberately throughout the scanning session. The participants were monitored using an eye-tracking system to ensure that they remained awake throughout the scan (Eyelink 1000, SR Research, Mississauga, ON, Canada).

### 2.3. Data preprocessing

Data preprocessing was performed using DPABI software (http://rfmri.org/dpabi) based on Statistical Parametric Mapping 12 (SPM12, http://www.fil.ion.ucl.ac.uk/spm/) on the MATLAB platform (MathWorks, Inc., Natick, MA). Due to signal equilibration, the first ten volumes were discarded. This was followed by slice timing and realignment with an appraisal of voxel-specific head motion. The head motion parameters were determined for each participant; those with movements above 3 mm translation and 3° rotation were excluded from further examination. A total of four participants were excluded due to excessive head movements. Next, functional scans were registered using T1 images and normalized to the Montreal Neurological Institute (MNI) template using DARTEL (Ashburner, 2007) at a resolution of 3 × 3 × 3 mm^3^. In total, seven participants were excluded due to low-quality image registration. Functional data were spatially smoothed using a 4-mm fullwidth half maximum (FWHM) Gaussian kernel to increase the signal-to-noise ratio. The signal was band-pass filtered (0.01–0.1 Hz). Finally, the nuisance signals (24 motion parameters, cerebrospinal fluid, and white matter signals) were removed from the time course of each voxel (Behzadi et al., 2007). We did not regress out the global signal to keep any additional information (Liu et al., 2017).

### 2.4. Brain network construction

**Fig. 1.**
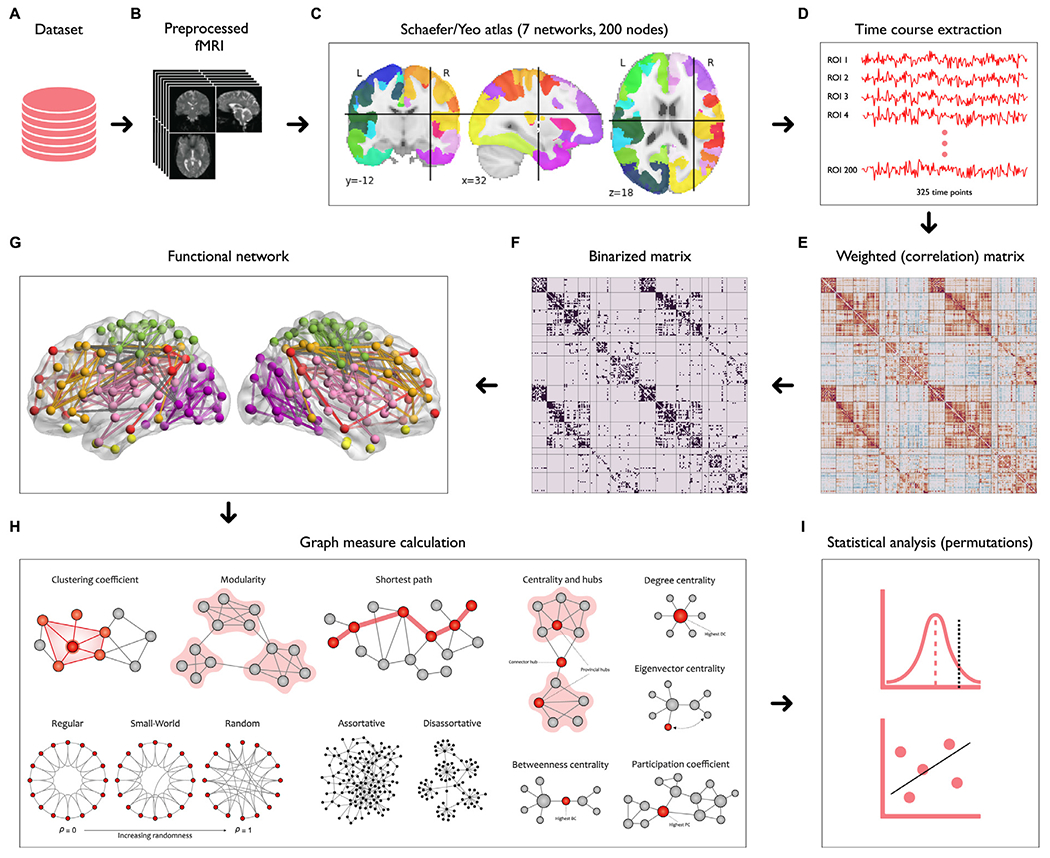
Schematic representation of our graph-based analysis. After preprocessing (B) the raw rs-fMRI data (A) and parcellating the brain into 200 regions of interest using Schaefer-Yeo atlas (C), corresponding time courses were extracted from each region (D) to compute the weighted correlation matrix (E). To reduce the complexity, a binary correlation matrix (F) and the corresponding functional brain network (G) were constructed. A set of global and local graph theory measures were then derived from these connectivity matrices (H). Finally, non-parametric statistics were applied to identify significant group means and correlations (I).

Our analysis pipeline is shown in Fig. 1. A large-scale brain network consists of a finite set of nodes (e.g., single neurons or anatomical brain regions) that are connected by edges (e.g., structural or functional connections between nodes). We specified the nodes by parceling the brain into seven systems/networks consisting of 200 cortical regions of interest (ROIs) from the Schaefer-Yeo atlas (Schaefer et al., 2018; Fig. 1C). In this atlas, each node is preassigned to one of the following systems/networks: visual, somatomotor, dorsal attention, salience/ventral attention, limbic, frontoparietal, or default mode. Then, the average BOLD time series across all voxels within each ROI were extracted separately (Fig. 1D). The connectivity between each pair of ROIs was then computed using Pearson’s correlation coefficient. The correlation values were converted into z-values using Fisher’s *r*-to-*z* transformation to improve the normality. At this stage, a symmetrical weighted connectivity matrix (adjacency matrix) with a size of 200 × 200 was constructed for each participant (Fig. 1E).

**Fig. 2.**
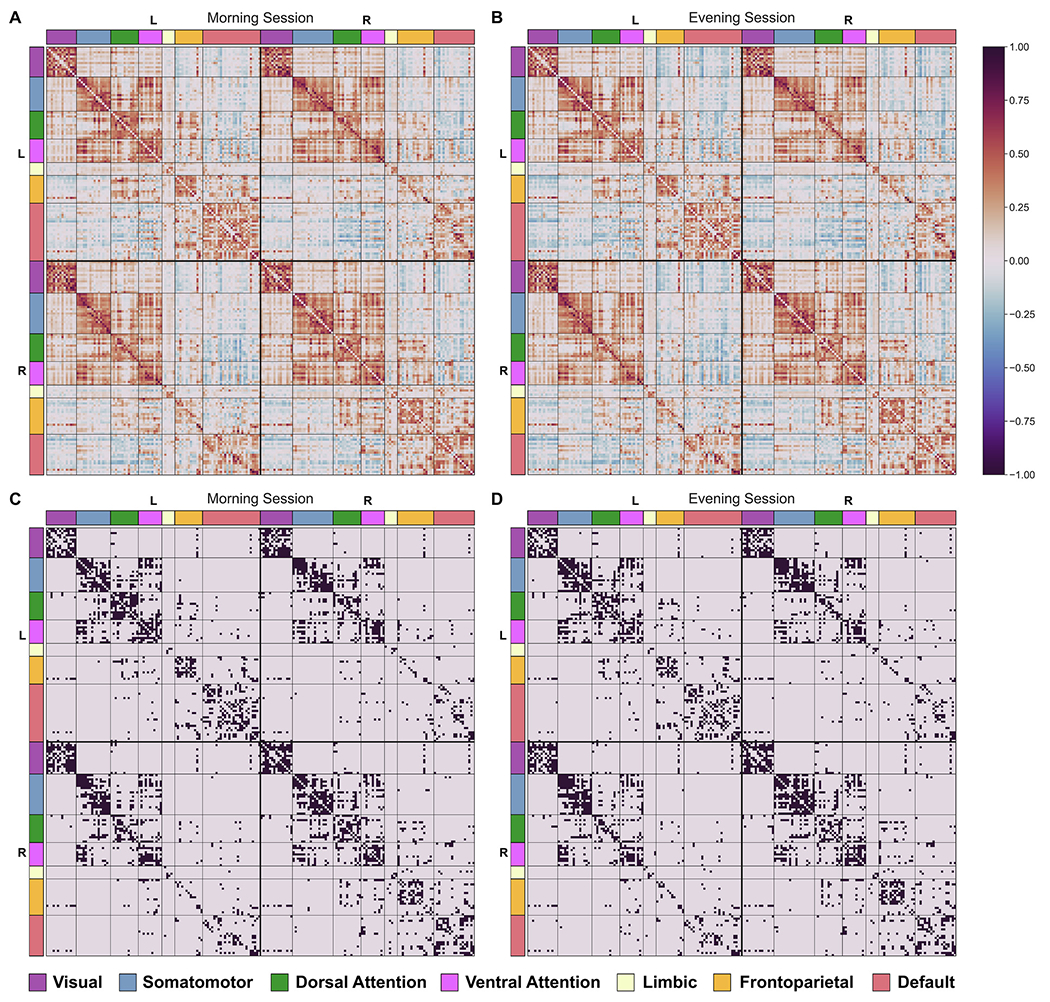
Weighted connectivity matrices (top) and binarized connectivity matrices (bottom, top 10% of strongest connections) for both morning (A and C) and evening (B and D) sessions (averaged across all participants in each session). The regions (nodes) are ordered according to which cognitive system they belong to.

To reduce the number of spurious connections in the fully weighted matrices (Power et al., 2011), we adopted a thresholding procedure based on network density to preserve the ratios of the strongest connections and remove the weaker links (van den Heuvel et al., 2017). This procedure leads to equal network density across all participants (i.e., an equal number of edges), crucial for comparing network topology within or between participants (Gamboa et al., 2014). The sparsity threshold used in this study ranged from 0.05 to 0.5 with an interval of 0.05 to prevent the creation of either disconnected or densely connected networks (Wang et al., 2020). This step was followed by binarizing the thresholded matrices to render the computational complexity more tractable (Fig. 1F). We used the absolute value of all correlations in weighted matrices for binarization. In this study, we used both the weighted and binary matrices depending on the type of analysis (i.e., global, mesoscale, local, or hub analysis) described in the following sections. Fig. 2 displays the average weighted and binary matrices across participants in the morning and evening sessions. This figure indicates the allocation of nodes to each of the Schaefer-Yeo systems by colored rectangle patches as tick labels along axes.

### 2.5. Computation of graph measures

#### 2.5.1. Global and local metrics

Using binary undirected matrices, we examined the topological features of functional brain networks for each subject across a range of cost thresholds at the global and local levels with the brain connectivity toolbox (BCT; Rubinov and Sporns, 2010) and the GRETNA toolkit (Wang et al., 2015). Table 3 provides mathematical definitions and descriptive explanations of each network statistic. Global metrics principally measure the functional segregation and integration of brain networks. Thus, we calculated global efficiency, mean clustering coefficient, characteristic path length, small-worldness, assortativity, and synchronization. Local network measures were calculated separately for each node (region) by examining the nodal centrality and density of network hubs (i.e., nodes with more than the average number of links). Hubs can be classified as either provincial or connector, which contain mostly local connections within a module or both local and long-range links that connect nodes in different modules, respectively. We calculated the most common local properties, including degree centrality, betweenness centrality, nodal clustering coefficient, nodal efficiency, and participant coefficient (Rubinov and Sporns, 2010).

#### 2.5.2. Mesoscale metrics

We utilized a multi-layer (or multi-slice) community detection algorithm (Mucha et al., 2010) to explore the modular structure of the resting-state brain networks across individuals. Each layer corresponds to an individual’s functional connectivity matrix (weighted matrix). This algorithm ensures that the community assignments (labels) are preserved across layers, thus making them comparable to each other. The multi-layer modularity function *Q* is generally initialized with two crucial parameters, structural resolution *γ* and interlayer coupling *ω* (see Table 3 for mathematical definitions). They tune the size of modules within each layer and the number of modules across layers, respectively. Although finding the optimal *ω* and *γ* is not straightforward, strategies for achieving reasonable solutions have been proposed (Tardiff et al., 2021). In this study, we first formed a 2D discrete parameter space inspired by previous studies (*γ* ∈ [0.5, 1.5] with a step size of 0.05; *ω* ∈ [0, 1] with a step size of 0.05). Then, we performed the modularity maximization procedure for all (*γ*, *ω*) combinations in the space and selected the corresponding parameter values with the highest *Q*, which resulted in *γ* = 1.2 and *ω* = 0.1. In each run, the multi-layer modularity function outputs the community labels in addition to *Q*, which are used to create the *module allegiance matrix*.

The module allegiance matrix (Figs. 4A and 4B) represents the fraction of layers in which two nodes belong to the same community (Bassett et al., 2015). To construct an allegiance matrix, we created a co-occurrence matrix (200×200) for each layer, wherein the *ij^th^* element is equal to 1 if the nodes *i* and *j* have a shared community label, and 0 otherwise. The average of all co-occurrence matrices across layers (62 layers per session) forms the allegiance matrix; thus, its elements range from 0 to 1. Based on the module allegiance matrix, we computed two network coefficients at the mesoscale called *recruitment* and *integration* (Bassett et al., 2015) to compare community structure between the target populations (see Table 3 for mathematical definitions).

### 2.6. Statistical tests

For global and local analyses, we applied a non-parametric permutation test (*p*-values were estimated from 30,000 permutations of group labels) in a mixed-design ANOVA to compare the mean differences between groups (considering the interaction effect) in which the within-subjects factor represented TOD, and the between-subjects factor represented chronotype (Anderson, 2001). For mesoscale and correlation analyses, we used one-way permutation tests to examine the difference between sessions (i.e., morning and evening) and detect correlations’ significance, respectively. This approach does not require distributional assumptions (Nichols and Holmes, 2002). A false discovery rate (FDR) correction was applied to all statistical tests (Benjamini and Hochberg, 1995).

## 3. Results

### 3.1. Global analysis

**Fig. 3.**
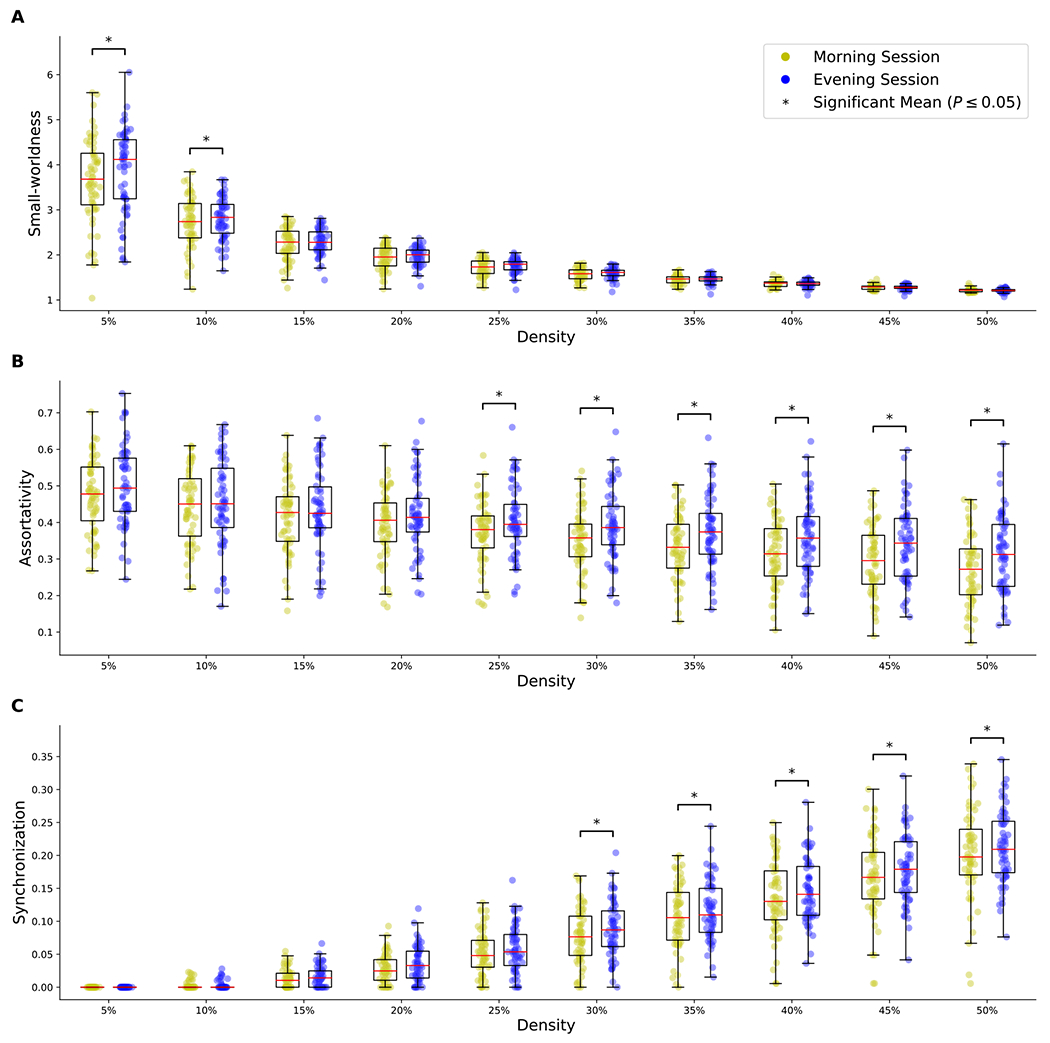
Differences in small-worldness (A), assortativity (B), and synchronization (C) between the morning and evening sessions at threshold values of 0.05 to 0.5 (p-values were computed using 30,000 permutations).

We compared FC between morning and evening sessions and found significant differences in small-worldness, network synchronization, and assortativity (Fig. 3). No statistical evidence of change was found in any other global measures. Small-worldness (Fig. 3A) decreased with higher network sparsity in both sessions. Compared with the morning session, results from the evening session revealed higher small-worldness at a sparsity of 0.05 and 0.1 (*p* < 0.05, FDR corrected), which did not differ between chronotypes. Assortativity (Fig. 3B ) and network synchronization (Fig. 3C) increased with higher network sparsity in both sessions. Our analysis revealed that assortativity and synchronization were significantly higher during the evening session than the morning session at a sparsity of 0.3 to 0.5 (*p* < 0.05, FDR corrected), with no differences between chronotypes.

### 3.2. Mesoscale analysis

**Fig. 4.**
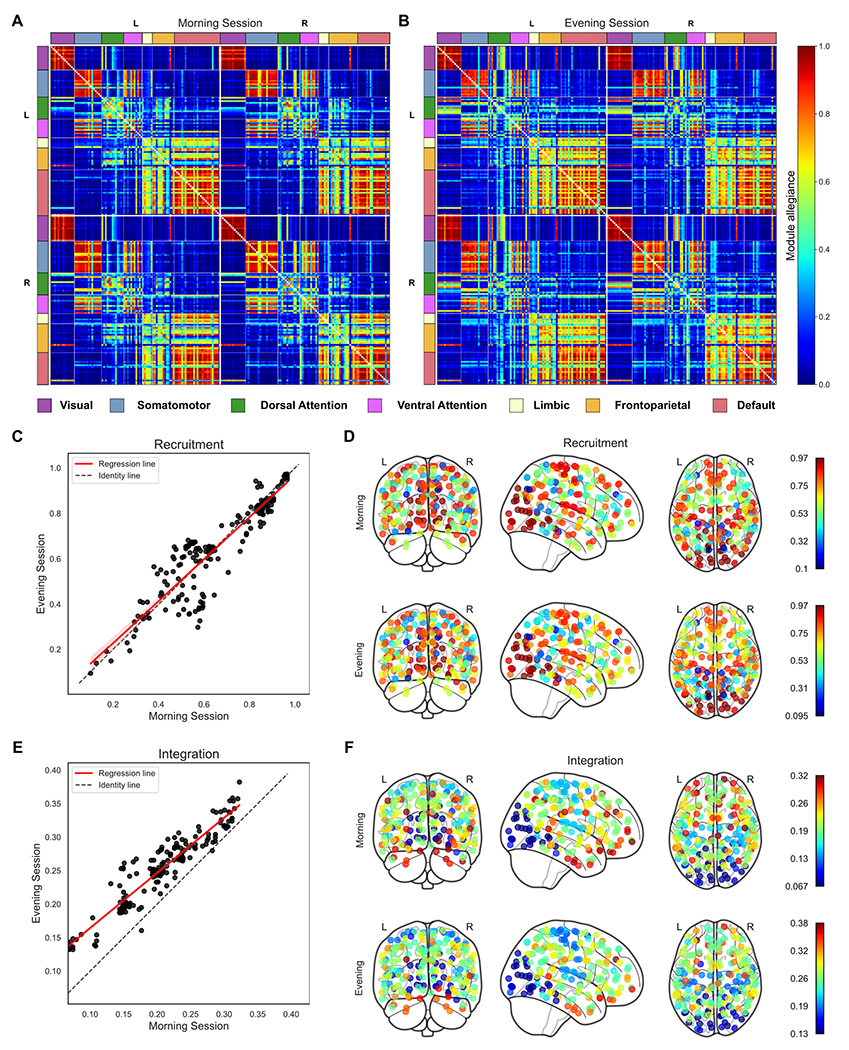
Recruitment and integration coefficients across brain regions. Panels (A) and (B) show the module allegiance matrices at the regional level for the morning and evening sessions, respectively, each representing the probability that two brain regions are assigned to the same community across individuals. The regions are arranged according to their belonging to 7 Schaefer-Yeo networks and for each hemisphere separately. In panels (C) and (D), we compared the recruitment coefficient between the sessions across brain regions, which are displayed by a scatterplot with the linear fit (*p* > 0.05, FDR corrected) and brain glasses, respectively. Similar plots, as for the recruitment, are shown for the integration coefficient in panels (E) and (F), where there is a significant increase in the evening compared to the morning (*p* < 0.05, FDR corrected). In general, as shown in (D) and (F), neighboring areas tend to have similar recruitment and integration coefficients, confirming the presence of cohesive large-scale structures in the brain.

We used a multi-layer (multi-subject) modularity framework (Mucha et al., 2010) to compare the community structure of the functional connectivity between sessions. Although most multi-layer modularity studies are about uncovering time-varying patterns, other applications such as the study of communities across subjects or task states have been performed (Betzel et al., 2019; Zamani Esfahlani et al., 2021). So, we assumed each layer as the weighted connectivity matrix of each individual in this study. By operating the multi-layer modularity function and taking the community labels, we created a module allegiance matrix (Bassett et al., 2015) per session (Figs. 4A and 4B). The module allegiance matrix displays how 200 brain regions and 7 Schaefer-Yeo networks/systems are cohesively engaged across individuals (Mattar et al., 2015). Then we extracted the recruitment and integration coefficients from allegiance matrices (Bassett et al., 2015) to compare the modular structure between the morning and evening groups. See Methods on how to build these metrics.

These coefficients allow the functional interplay among brain regions and predefined/static functional systems to be quantified. Recruitment measures how a region is recruited to its own system across individuals, and integration measures the extent to which a region is integrated with other systems across individuals. Each row/column of the allegiance matrix corresponds to a brain region whose average values inside and outside of its static system yield the recruitment and integration coefficients of that region, respectively. According to Figs. 4A and 4B, the warm block-like patterns along the diagonal of each quadrant in allegiance matrices confirm that predefined systems generally tend to be recruited than integrated with other systems across individuals (Cole et al., 2014).

Fig. 4C compares the recruitment coefficients of the 200 brain regions between the morning and evening sessions as a scatterplot with the linear regression fit (red line). We applied a permutation test by shuffling the group labels to see if the regression line differed significantly from the identity line. As can be seen, the recruitment results were consistent with the null (*p* > 0.05, FDR corrected). The recruitment coefficients are also plotted on top of the brain glass schematics in Fig. 4D for both sessions. This plot shows how brain regions differed in recruitment from their predefined/static system across participants. A similar scatterplot and brain glass for the integration coefficient are also shown in Figs. 4E and 4F, respectively. Contrary to recruitment results, these plots confirm that the difference in integration coefficients between the morning and evening sessions was inconsistent with the null (*p* < 0.05, FDR corrected). Notably, we found that the brain areas in the evening session were more integrated with the regions of other systems than in the morning.

**Fig. 5.**
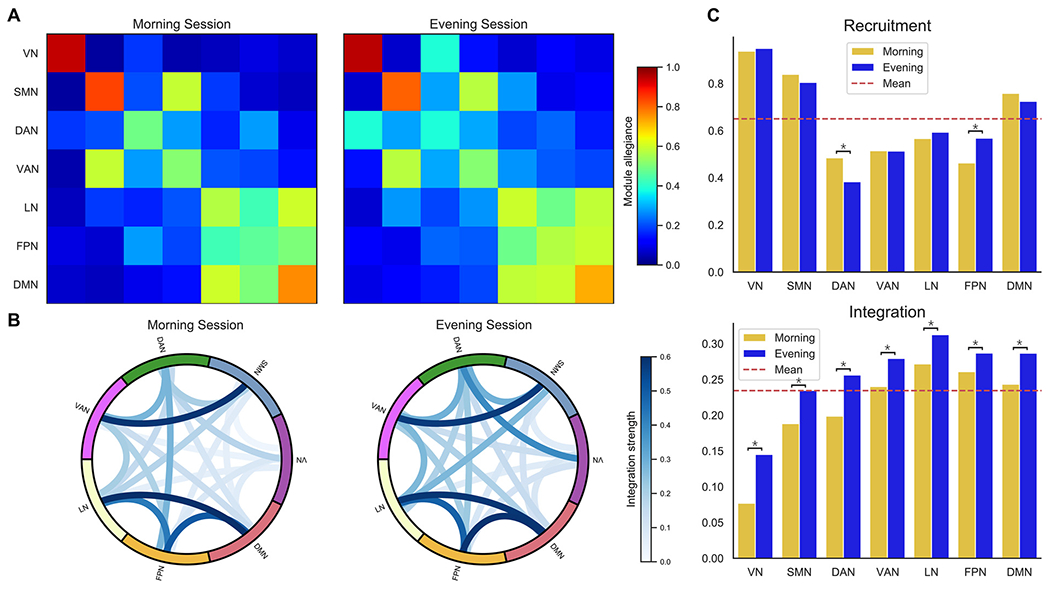
Recruitment and integration coefficients across large-scale brain systems. Panels (A) shows the module allegiance matrices at the system level for the morning and evening sessions, each representing the engagement of predefined systems in the whole brain organization across individuals. (B) Comparing network integration between morning and evening sessions, where each edge represents the integration between a pair of systems. Color intensity indicates edge strength, ranging from light to dark blue. (C) Comparing the system’s recruitment and integration between morning and evening sessions. Significant differences are noted with asterisks (*p* < 0.05, FDR corrected). Abbreviations: VN – visual network; SMN – somatomotor network; DAN – dorsal attention network; VAN – ventral attention network; LN – limbic network; FPN – frontoparietal network; DMN – default mode network.

In addition to regionally studying the brain as shown in Fig. 4 (i.e., thoroughly investigating the role of each region, both within its network and into other networks), a coarser granularity of brain interactions at the systemic scale across individuals could be explored by smoothing out regional information (Mattar et al., 2015). Therefore, we defined the system-level module allegiance matrix so that its *kl^th^* element was computed as the average of the values of all pairs of regions between systems *k* and *l* (including *k* = *l*) from the regional-level module allegiance matrix (i.e., the mean of squares gridded with white lines). We then merged the information from both hemispheres, resulting in a 7 × 7 allegiance matrix that exhibits how regions from large-scale systems are engaged in the functional brain network (Fig. 5A). Next, using these allegiance matrices derived from both morning and evening sessions, we calculated and compared their recruitment and integration coefficients for systems instead of regions. For a given system, the recruitment coefficient is the probability that any region of that system has the same community label as the other regions within that system. Simply put, the diagonal elements of the allegiance matrix correspond to the recruitment coefficients of the large-scale systems. Also, the integration coefficient between each pair of systems is the average probability that regions in one system share the same community label as regions in another system (off-diagonal elements of the allegiance matrix). See Table 3 for detailed mathematical definitions.

Fig. 5A shows that the systems differed in their strength of network recruitment and integration in both morning and evening sessions. Some systems were more consistently recruited across participants, such as the visual and somatomotor networks. At the same time, some were less recruited, such as the dorsal attention, ventral attention, and frontoparietal networks (see diagonal elements). For a more transparent illustration of the integration measure, we also created a chord diagram per session (Fig. 5B) using off-diagonal elements of the allegiance matrices, in which the edges represent the network integration between the brain systems. Among all systems in both sessions, the visual network was less integrated with other networks (also less in the morning than in the afternoon). We also found that the limbic, frontoparietal, and default networks tended to be well integrated among themselves. Finally, we observed that the somatomotor and ventral attention systems were well integrated. This graphical representation helps to understand better the complex patterns of integrations in a heterogeneous set of large-scale systems.

We also compared each system’s recruitment and average integration (to all other systems) between the morning and evening sessions in Fig. 5C. Thus, for each coefficient and in each system, we performed a permutation test in which the group labels were shuffled repeatedly to check whether the difference in sessions was in line with the null or not. Significant differences are marked with an asterisk above each pair of bars (*p* < 0.05, FDR corrected). We found that all systems were more integrated with most other systems in the evening than in the morning. While there were significant differences for the recruitment coefficient only in the dorsal attention and frontoparietal systems, the former decreased and the latter increased during the day. Despite all these variations, the systems generally tended to be more recruited than integrated with other systems across individuals, reflecting the cohesive nature of large-scale systems in the brain (Mattar et al., 2015).

### 3.3. Local analysis

**Fig. 6.**
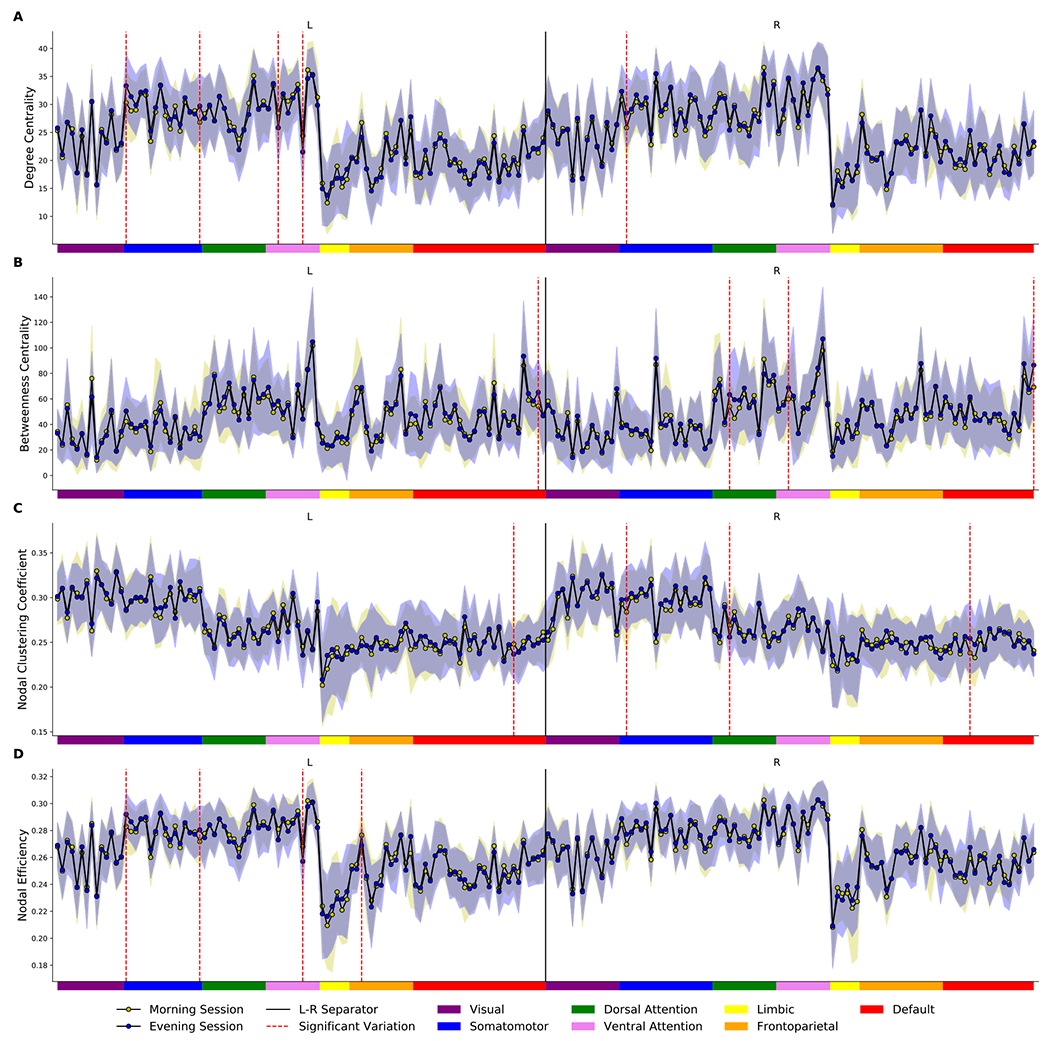
Area under the curve in the morning and evening sessions for degree centrality (A), betweenness centrality (B), clustering coefficient (C), and nodal efficiency (D) in all 200 brain regions of interest. Each node in either the left or right hemisphere is labeled with a color that is matched to Schaefer-Yeo 7 network parcellation. Significant diurnal changes are represented by dashed red lines.

Table 4 summarizes brain regions that exhibited significant differences between the morning and evening sessions (first factor) and between the “lark” and “owl” participants (second factor), based on their nodal/local properties in more than half of the network sparsity. As shown in Table 4, the measures of degree, betweenness centrality, clustering coefficient, and nodal efficiency were computed for group comparisons. Most of these differences involved regions and their homotopic partners in the opposite hemisphere. No significant differences in participation coefficient and nodal shortest path were found for either factor (*p* > 0.05, FDR corrected). The results of the area under the curve (AUC) analysis for the degree, betweenness centrality, clustering coefficient, and nodal efficiency for all 200 brain regions are presented in Fig. 6. The AUC was calculated for each metric to provide a scalar not contingent on a particular threshold value (Wang et al., 2009; Zhang et al., 2011).

According to Table 4 and Fig. 6, compared with the morning session, the evening session showed a significantly higher nodal degree in the somatomotor network and in areas such as the bilateral superior temporal gyrus, and postcentral gyrus as well as a decreased degree of centrality in the left ventral attention network and regions such as the supramarginal and middle frontal (*p* < 0.05, FDR corrected). Similar results were obtained for nodal efficiency, together with a significant reduction in FC throughout the day in the left angular gyrus (*p* < 0.05, FDR corrected). Betweenness centrality analysis also showed a substantial increase in the evening session compared with the morning session in areas such as the bilateral precuneus as well as the right angular and right supramarginal gyri (*p* < 0.05, FDR corrected). Finally, the nodal clustering coefficient was higher in the evening session than in the morning session in the right superior temporal gyrus, while this value was lower in the left superior frontal gyrus and right angular gyrus later in the day (*p* < 0.05, FDR corrected).

**Fig. 7.**
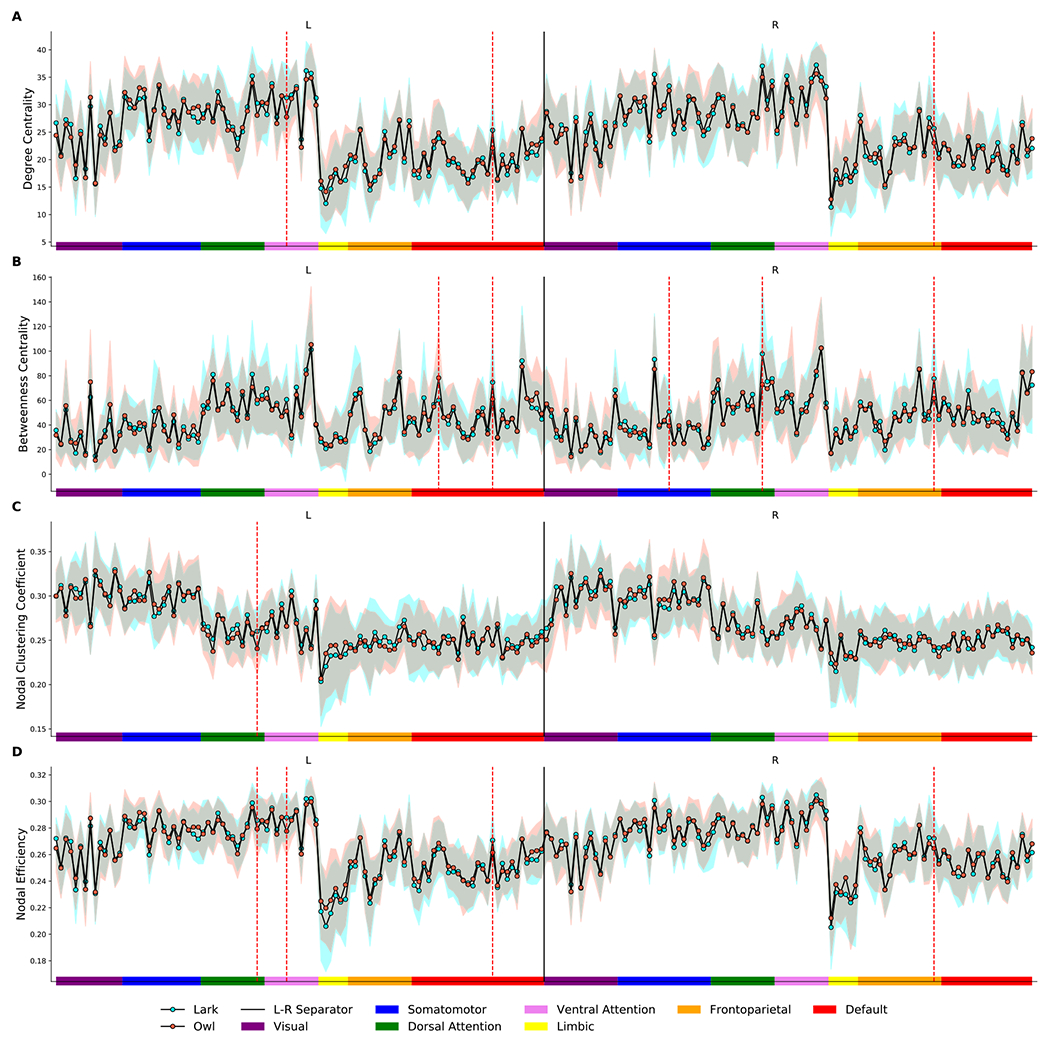
Area under the curve for the “lark” (morning-type) and “owl” (evening-type) participants for degree centrality (A), betweenness centrality (B), clustering coefficient (C), and nodal efficiency (D) in all 200 brain regions of interest. Each node in either the left or right hemisphere is labeled with a color that is matched to Schaefer-Yeo 7 network parcellation. Significant diurnal changes are represented by dashed red lines.

In chronotype analysis (Fig. 7), degree centrality and nodal efficiency underwent a significant decrease in the bilateral dorsal anterior cingulate cortex and left insular cortex in the ET participants compared with the MT (*p* < 0.05, FDR corrected). Also, a comparison of the clustering coefficient and nodal efficiency characteristic of the left superior frontal gyrus showed significantly lower values in the ET than in the MT (*p* < 0.05, FDR corrected). Finally, the betweenness centrality in areas that included the bilateral dorsal anterior cingulate gyrus and right precentral gyrus showed significantly lower values in FC among the ET subjects compared to the results from the MT group (*p* < 0.05, FDR corrected).

### 3.4. Hub analysis

**Fig. 8.**
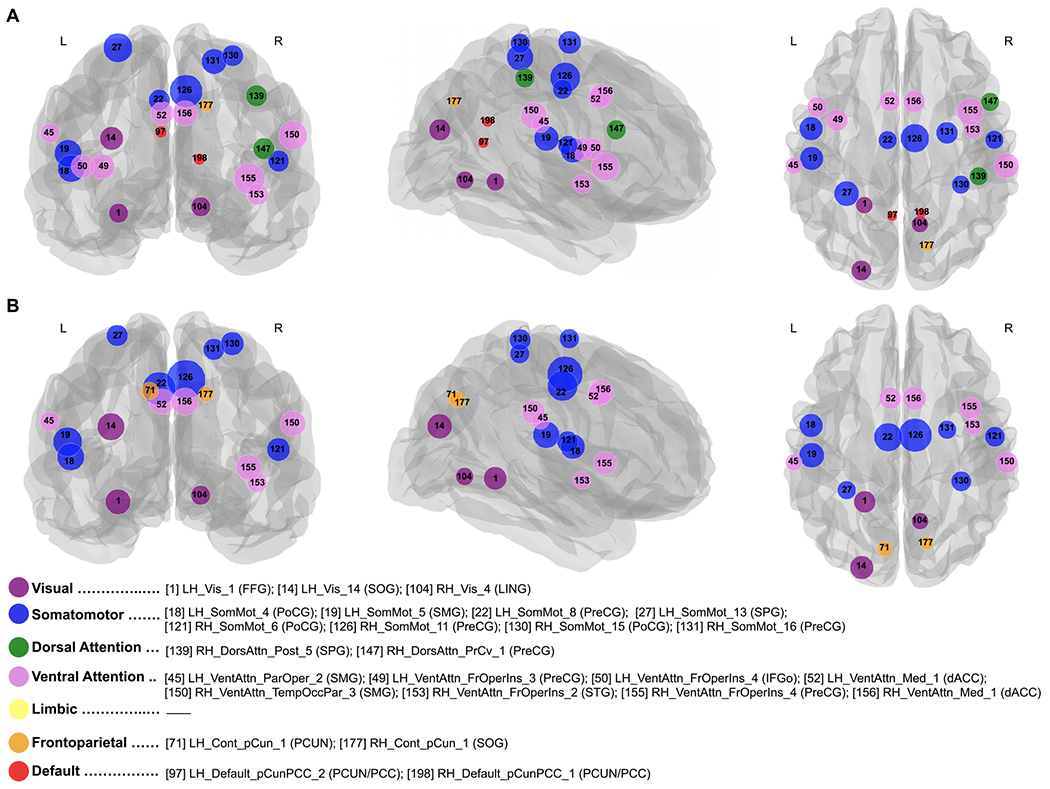
Hubs (highly connected regions) in the morning (A) and evening (B) sessions were determined at a sparsity of 0.05. Regional information is provided in Appendix Table A1. Abbreviations: *LH/RH* – left/right hemisphere; *Vis_[i]* – *i*th segment of the Visual Network; *SomMot_[i]* – *i*th segment of the Somatomotor Network; *DorsAttn_Post_5* – fifth segment of the posterior Dorsal Attentional Network; *DorsAttn_PrCv_1* – first segment of the precentral ventral Dorsal Attentional Network; *SalVentAttn_ParOper_2* – second segment of the parietal operculum Ventral Attention Network; *SalVentAttn_FrOperIns_[i]* – *i*th segment of the frontal operculum insula Ventral Attention Network; *SalVentAttn_Med_1* – first segment of the medial Ventral Attention Network; *SalVentAttn_TempOccPar_3* – third segment of the temporal occipital parietal Ventral Attention Network; *Cont_pCun_1* – first segment of the precuneus Control Network; *Default_pCunPCC_[i]* – *i*th segment of the precuneus posterior cingulate cortex Default Network; *FFG* – fusiform gyrus; *SOG* – superior occipital gyrus; *LING* – lingual gyrus; *PoCG* – postcentral gyrus; *SMG* – supramarginal gyrus; *PreCG* – precentral gyrus; *SPG* – superior parietal gyrus; *IFGo* – inferior frontal gyrus (pars opercularis); *dACC* – dorsal anterior cingulate cortex; *STG* – superior temporal gyrus; *CUN* – cuneus; *PCUN* – precuneus; *PCC* – posterior cingulate cortex. Labels from the Yeo and Schaefer Atlas are available here.

**Fig. 9.**
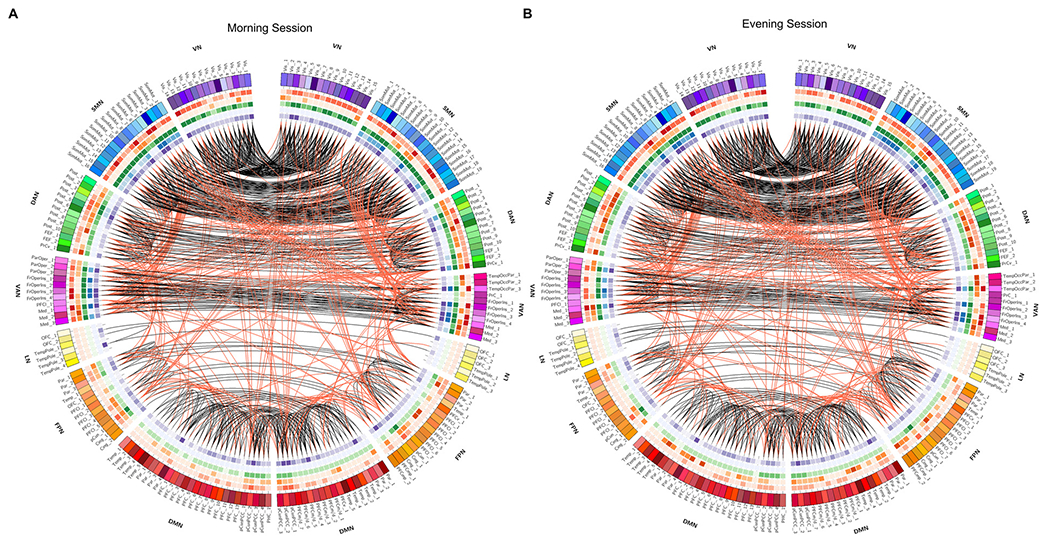
The mean connectogram across all participants in the morning (A) and evening (B) sessions at a thresholding value of 0.05. Parcellated elements within the outermost circle include the 200 Schaefer/Yeo brain areas marked with a unique RGB code that has been associated with one of the predefined modules in each hemisphere. The outer circle circumscribes a set of five inner circular heatmaps that were created to represent the values of five different centrality measures. The range of colors for each metric represents the minimum to the maximum values. Toward the center, these measures are degree centrality, participation coefficient, K-coreness centrality, eigenvector centrality, and PageRank. The values of all measurements and the functional connections in each connectogram are derived from the mean of all participants in the corresponding session. The red and black curves indicate the functional connections between and within modules, respectively. An unambiguous abbreviation scheme was created to label each parcellation, as summarized in Appendix Table A1. Abbreviations: VN – visual network; SMN – somatomotor network; DAN – dorsal attention network; VAN – ventral attention network; LN – limbic network; FPN – frontoparietal network; DMN – default mode network.

In this subsection, using a pre-determined modular classification that includes the visual, somatomotor, dorsal attention, ventral attention, limbic, frontoparietal, and default mode network (Yeo et al., 2011), we identified network hubs (connector or provincial) for the morning and evening sessions, as well as for the MT and ET groups. In this regard, we found changes in hub organization across the TOD (Fig. 8). No significant differences were detected with respect to chronotype. The results presented in Fig. 8 are based on the average connectivity matrix (across all individuals for each scanning session). For ease of visualization, we selected a network density of 0.05. As can be seen, the identified hubs nearly overlap with one another in the morning and evening sessions, except for changes in the left frontal operculum insula (LH_SalVentAttn_FrOperIns_3, 4), right superior parietal gyrus (RH_DorsAttn_Post_5), right precentral ventral gyrus (RH_DorsAttn_PrCv_1), left precuneus (LH_Cont_pCun_1), and bilateral posterior cingulate cortex/precuneus (Default_pCunPCC_1, 2). We found that the somatomotor network contains more hubs than any of the other networks in both morning and evening sessions. Notably, its hubs are both provincial (i.e., within modular connections) and connector (i.e., between modular connections), at nearly the same ratio. In contrast, hubs in the ventral attention network are connector, while hubs identified in the visual network were mostly provincial. To examine connections among all regions and to identify hub types (i.e., connector or provincial), connectograms of both sessions were created using Circos software (Krzywinski et al., 2009). The results are illustrated in Fig. 9.

### 3.5. Correlation analysis

**Fig. 10.**
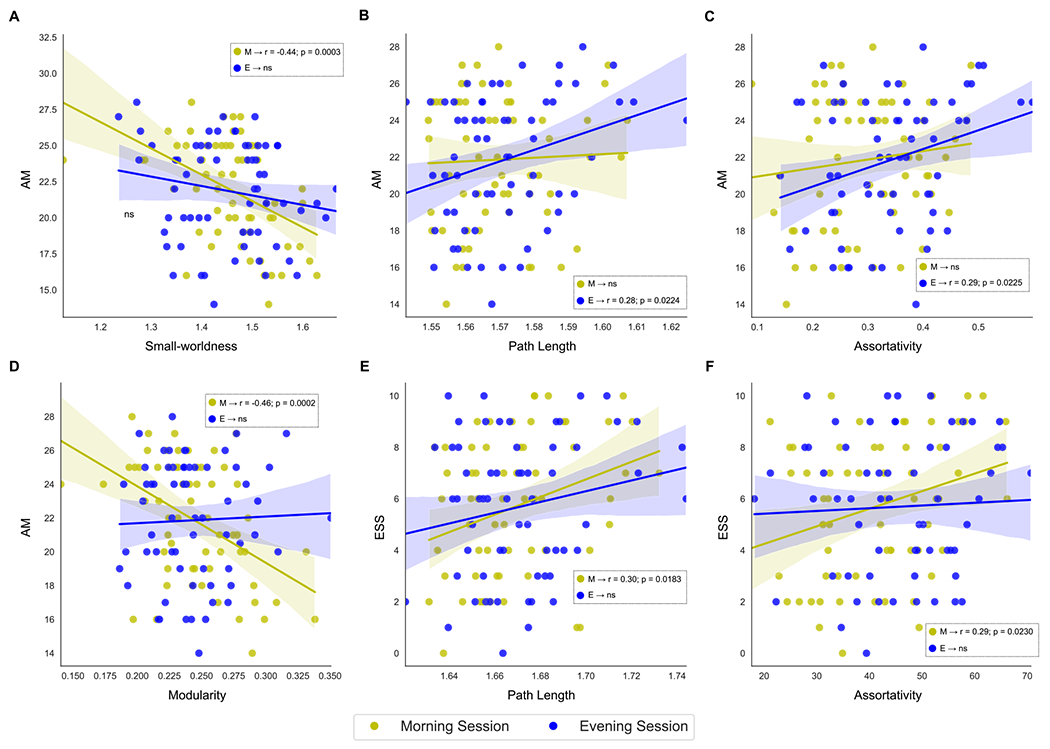
Significant associations between the global measures and questionnaire variables (AM, ESS, and ME scores): AM and small-worldness (A), AM and path length (B), AM and assortativity (C), AM and modularity (D), ESS and path length (E), and ESS and assortativity (D). Yellow and blue colors represent the scatterplots for morning and evening sessions, respectively. Statistically significant correlations are indicated in each panel.

A correlation analysis was performed to determine whether global and nodal measures throughout the day were significantly associated with variables of interest (e.g., ME scale, AM scale, and ESS) across participants while controlling for the differences among the covariates of no interest (e.g., age, sex, and clinical variables). The ME, AM, and ESS scores represent an individual’s chronotype preference, the strength of this preference, and the degree of sleepiness during the day, respectively. Overall, the number of significant associations was greater in the morning session than in the evening session. From a global perspective (Table 5 and Fig. 10), correlation analysis revealed significant negative associations between AM scores and both small-worldness and modularity in the morning session (*p* < 0.05, FDR corrected). We also found significant positive correlations between ESS and average path length and assortativity in the morning session and positive correlations between AM score and path length and assortativity in the evening session.

**Fig. 11.**
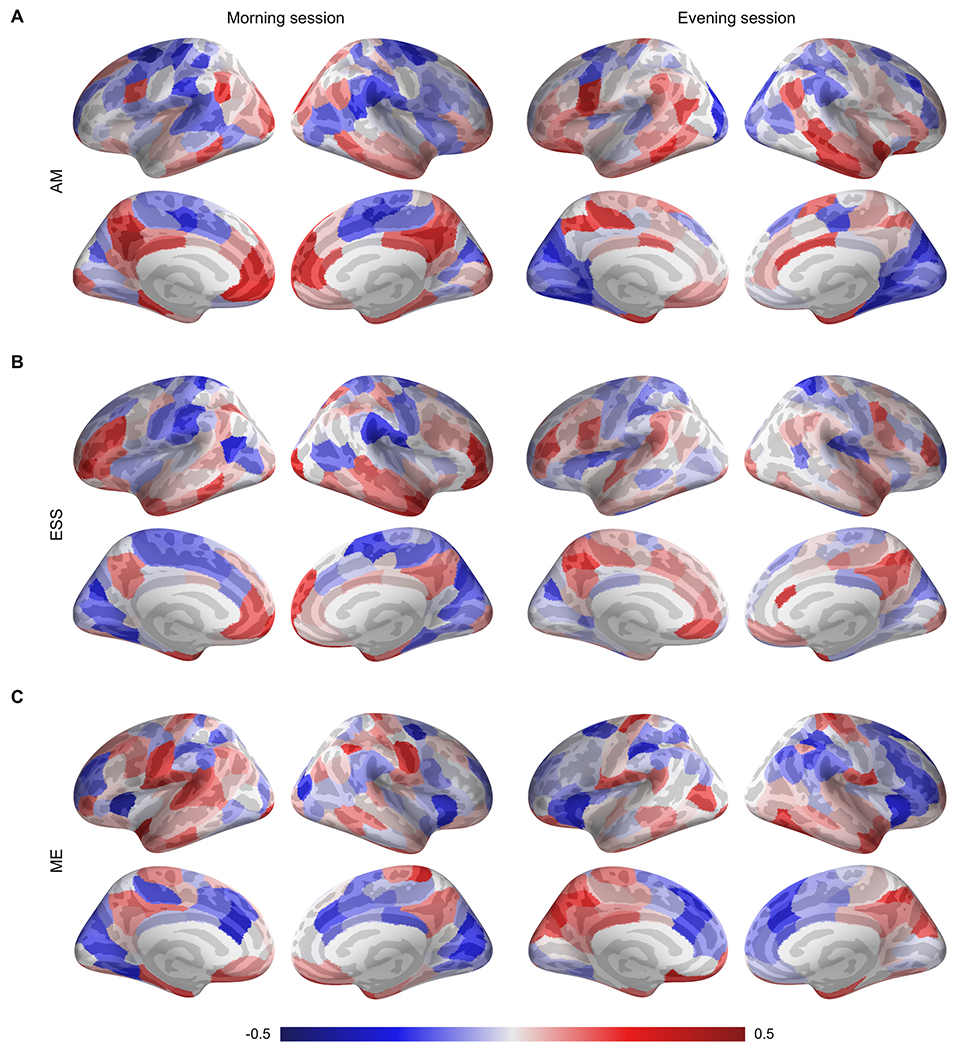
Correlation analysis between the nodal centrality of brain regions and the questionnaire variables: AM (A), ESS (B), and ME (C). Nodes are colored according to the magnitude of the correlation.

From the nodal perspective, we found significant correlations between the degree centrality of various brain regions in both hemispheres and these subjective indicators (ME scale, AM scale, and ESS), mostly across the morning scanning session (Table 6 and Fig. 11). In the morning session, significant negative correlations were found between AM scores and areas within the default network, including the left rostral anterior cingulate gyrus (Default_PFC_6), left precuneus (Default_pCunPCC_2, 4), right medial prefrontal cortex (Default_PFCm_4), and right posterior cingulate cortex (Default_pCunPCC_2); between ESS and the left pole of the superior temporal gyrus (Limbic_TempPole_3), the right lateral fronto-orbital gyrus (Cont_PFCl_1), and the right pole of the middle temporal gyrus (Default_Temp_1); and between ME scores and the left postcentral gyrus (SomMot_4) and the left pole of the superior temporal gyrus (Limbic_TempPole_4). Significant positive associations were found between AM scores and the bilateral precentral gyrus (left DorsAttn_FEF_1 and right SomMot_11) and between ME scores and the bilateral insula (SalVentAttn_FrOper_2 and Cont_PFCv_1), the left anterior cingulate gyrus (Default_PFC_8), and the right precentral gyrus (DorsAttn_FEF_1).

During the evening session, we identified significant positive associations between AM scores and the left middle occipital gyrus (Vis_11), right fusiform gyrus (Vis_2), and right superior occipital gyrus (Vis_14), as well as negative correlations between AM scores and the left precentral gyrus (DorsAttn_PrCv_1), bilateral lateral fronto-orbital gyrus (Limbic_OFC_1, 2), and right middle temporal gyrus (Default_Temp_4). No significant correlations were found between ESS and ME scores and the degree centrality of these brain regions.

## 4. Discussion

The current study, used rs-fMRI, a chronotype-based paradigm, and graph theory to examine the diurnal fluctuations of whole-brain connectivity architecture in 62 young, healthy participants. The study revealed meaningful information regarding the topological variations of the brain network during the day and organizational differences between the “lark” and “owl” groups, as well as associations of graph theory metrics with selected variables of interest (i.e., ME, AM, and ESS scores).

The main results can be summarized as follows: (1) Among the global measures, there was a significant increase in small-worldness, assortativity, and network synchronization in the evening session over the morning session (*p* < 0.05, FDR corrected). However, there was no compelling evidence of changes in any of the global metrics in chronotype (i.e., between MT and ET participants). (2) Mesoscale analysis showed higher brain systems/regions integration among themselves in the evening session. (3) Local graph measures varied during the day and between the two chronotypes, predominantly across the somatomotor, attention, and default-mode networks. (4) Analysis of the hubs revealed that the somatomotor network was the densest area of the brain in both sessions, but more so during the evening session, including both provincial and connector types, whereas hubs in the ventral attention network and visual network were primarily connector and provincial, respectively. (5) Correlation analysis revealed significant associations between the variables derived from the questionnaires (ME, AM, and ESS) and the nodal characteristics of several brain regions in both scanning sessions, most of which were associated with the morning session.

### 4.1. Diurnal variations in the brain network as a whole (global properties)

A small-world network is an intermediary between a random and a regular grid that contains many short-range connections alongside a few long-range shortcuts (Watts and Strogatz, 1998). There are other ways to define a small-world network (e.g., by considering the physical length of connections), all of which suggest mathematically that small-world networks share relatively high transitivity and small mean geodesic distance (i.e., shortest path) between nodes. This property strikes an optimal balance between network integration and segregation (Bassett and Bullmore, 2006; Rubinov and Sporns, 2010). In our analysis of rs-fMRI data, we found high values of small-worldness for both scanning sessions (small-world networks generally have an *σ* of at least 1), albeit with significant superiority at highly sparse networks in the evening compared with the morning session. In another study, Anderson et al. (2017) explored how TOD affects functional brain networks in older healthy adults, and they found no topological changes in small-worldness during the resting state. It is noteworthy that the significant changes that we found occurred only at lower densities, and—as in Anderson et al. (2017), who considered a sparsity range of 0.1 to 0.7—we found no significant difference at rest during the day at densities above 0.1.

However, the small-worldness provides little information on the actual network organization, and comparing its values is not straightforward because this measure does not follow a linear relationship. A review study (Mark D. Humphries and Gurney, 2008) shows that when the wiring structure of a regular network is gradually moving towards an utterly random architecture, the small-worldness, *σ*, first increases to reach a peak, and then decreases. The nonlinear nature of the small-worldness complicates the assessment of two given networks as they may have similar values falling on either side of the peak, while their topological structure is different (i.e., one side could be more like a regular network while the other side could be more like a random network). To further elucidate the small-worldness, we performed a system-level analysis to see how communities/modules were organized in the network for both sessions (see mesoscale analysis in Results). Therefore, we found that the daily increase in small-worldness, primarily at lower densities (global analysis), was due to the higher tendency of large-scale networks to be integrated with one another in the evening session (mesoscale analysis). Collectively, these findings reflect a more efficient topology of the information flow—due to the slight addition of randomness—in the evening session than in the morning session.

Furthermore, our findings revealed an increase in network assortativity over the course of the day, which is a finding that overlaps with our previous results (Farahani et al., 2019a). Increased assortativity is related to a higher propensity for a node to connect to other nodes with the same or a similar degree (Newman, 2003; Foster et al., 2010), thereby increasing the likelihood that a nearby hub will be capable of supporting a faulty node. Finally, the results of network synchronization, which is a measurement used to assess how well all nodes oscillate in the same wave pattern, were not consistent with the findings reported by Barahona and Pecora (2002). They showed that in networks of low redundancy, small-worldness results in higher synchrony than what is found in standard deterministic graphs, random graphs, and ideal constructive schemes. We discovered that small-worldness decreased and synchronization increased in morning and evening sessions as we moved from low to high sparsity. Notably, network synchronization was significantly higher during the evening scanning session than in the morning session. Overall, the findings of all global measures indicate that brain network organization varied throughout the day, a determination that might be associated with increased brain function and interaction from morning to evening.

### 4.2. Diurnal variations in the community structures (mesoscale properties)

Higher integration of DMN, limbic system, and frontoparietal regions in the evening session, revealed in the current study, has been previously proven to be associated with deliberate (not spontaneous) mind-wandering (Golchert et al., 2017). The above state of mind is thought to reflect cognitive control. Other studies indicate that individuals with higher control are better at modulating mind-wandering under task demands, presumably making it more efficient (Smallwood and Andrews-Hanna, 2013; Smallwood and Schooler, 2015). Interestingly, according to our different results, the stronger the chronotype, the more integrated neural networks are in the evening. The above results suggest that those evening hours benefit people with extreme chronotypes. The high predominance of spontaneous over deliberate mind-wandering is usually related to attention deficits (Seli et al., 2015b) and higher reactivity to inner experiences (Seli et al., 2015a). Moreover, the frontoparietal control network has been proven to include two separate subsystems where FPNa is interconnected with DMN and FPNb with DAN. As a result, the former part is highly associated with introspective processes, while the latter is related to the overall processing of the external stimuli (Dixon et al., 2018). Furthermore, somatomotor and ventral attention networks, which also showed higher integration in our study, are believed to be associated with sustained attention (Mitko et al., 2019). Overall, our mesoscale findings on network integration are congruent with other results in the study, pointing out a more prominent role of the frontoparietal regions in executive control during the evening hours (Dixon et al., 2018).

We also observed significant variation in the strength of network recruitment associated with the time of the day. The most striking example was the increased recruitment of frontoparietal systems during the course of the day. The above structure is believed to be associated with cognitive control and subsequent working-memory storage (Dormal et al., 2012); therefore, results from the current study may be related to the accumulation of information during the day. Notably, FPNb, which is interconnected with DAN, is associated with processing abstraction, monitoring, and manipulating sensorimotor contingencies (Dixon et al., 2018). Current results revealing increased recruitment in the FPN and decreased recruitment in the DAN are fascinating and deserve further investigation in the future.

### 4.3. Nodal/local changes affected by time of day

In this study, the somatomotor, attention, and default mode networks experienced the largest quantity of topological variations among brain networks during the day. Moreover, the study of hubs in the exact same networks was proved to host several densely connected nodes, which affect brain functional integration and segregation.

In the somatomotor network, resting-state findings from both morning and evening sessions revealed higher within-network connectivity than other brain networks and higher between-network connectivity, particularly with the ventral attention network. Moreover, both connectivity and hub analyses indicated that the somatomotor network contained the most highly connected areas in the brain, mainly during the evening session. We also found significantly higher FC in the bilateral superior temporal and left postcentral gyri as waking time increased. Consistent with our results, similar changes in the neural response across TOD were previously reported in other rs-fMRI studies (Anderson et al., 2014; Fafrowicz et al., 2019; Jiang et al., 2016; Maire et al., 2018), as well as in magnetoencephalography (MEG) studies evaluating oscillatory activity at rest and during a finger-tapping task (Wilson et al., 2014). On the contrary, conflicting results were presented in a morphometric study by Trefler et al. (2016), who discovered a significant decrease in cortical thickness as a function of TOD across the lateral surfaces of the left frontal, temporal, and parietal lobes. Nevertheless, findings from the current study provide insight into how changing functional activity in sensorimotor networks is associated with the course of the day. The increase of FC in somatomotor regions indicates that neural synchronization is enhanced in these areas.

Ventral and dorsal attention networks are believed to be involved in stimulus-driven and goal-directed attention, respectively (Vossel et al., 2013). Both connectivity and hub analyses revealed the ventral attention system to be the second most densely connected network, after the somatomotor areas. The above results were primarily observed during the morning session, focusing on the frontal operculum insula and dorsal anterior cingulate gyrus. Moreover, the study confirmed decreasing FC throughout the day within ventral areas such as the left supramarginal gyrus and left middle frontal gyrus (dorsal prefrontal cortex)—a finding that is in line with previous studies (Anderson et al., 2017; Jiang et al., 2016; Vandewalle et al., 2009). Other changes were also observed in the right dorsal attention areas, such as the angular and supramarginal gyri.

In addition, the current study revealed the default mode network (DMN) to be involved in relatively high neural activity at the resting state during both sessions, particularly in the posterior cingulate cortex, where it was identified as a hub. However, this activity was higher in the evening than in the morning session. The DMN consists of functionally-connected and specialized neural units, contributing to many cognitive functions (Andrews-Hanna et al., 2007; Mayer et al., 2010; Raichle et al., 2001). Moreover, the posterior cingulate cortex plays a crucial role in meditating intrinsic activity through the DMN (Fransson and Marrelec, 2008). Notably, some areas within the DMN were found to be prone to variations in their connectivity profiles across the day, such as the bilateral posterior cingulate cortex, precuneus, angular gyrus, superior temporal gyrus, and left superior frontal gyrus, a finding that is consistent with the results from previous rs-fMRI studies (Facer-Childs et al., 2019a; Fransson, 2005; Jiang et al., 2016; Ku et al., 2018; Lunsford-Avery et al., 2020; Orban et al., 2020; Raichle et al., 2001; Shannon et al., 2013).

Further studies reported a rhythmic FC pattern of the DMN during the day that peaked in the morning and declined during the afternoon (Blautzik et al., 2013; Hodkinson et al., 2014). It is believed that diurnal changes in these areas indicate the functional coordination of spatially disparate gyri (Jiang et al., 2016). Considering the findings in this study, total DMN neural activity decreased during the day; however, the FC increased in hub regions such as the posterior cingulate cortex and precuneus. Both the posterior cingulate cortex and precuneus are believed to be highly affected by decreased consciousness, which could explain the lower FC in the morning hours (Luppi et al., 2019). However, the results from the whole network are consistent with reports on sleep inertia, which is associated with increased DMN functional connectivity shortly after awakening (Vallat et al., 2019). This finding may suggest compensatory mechanisms of the mentioned areas to balance neuronal interactions, such as coupling or decoupling within DMN subregions. It could perhaps redouble efforts to increase the adaptability of the network under the continued wakeful condition throughout a day.

### 4.4. Nodal/local changes affected by chronotype differences

The current findings prove that the frontoparietal and dorsal attention networks underwent the most topological changes between earlier and later chronotypes. However, hub analysis did not show a statistically significant difference between the two groups. In the present study, participants with ET chronotypes revealed less neural activity and less network integration of the frontoparietal network (or central executive network) than the MT group, particularly in the dACC parcel. Consistent with our results, Horne and Norbury (2018) reported a significant reduction in FC between the dACC and amygdala in the latter chronotype, which they believed led to impaired emotion regulation. Overall, these findings could explain the role of chronotype in the interaction between the alerting functions and executive control networks (Martínez-Pérez et al., 2020).

### 4.5. Correlation between the network properties and subjective variables

The strength of the chronotype preferences was negatively correlated with small-worldness and modularity in the morning session. The graph measures presented are believed to be responsible for the integration of large-scale brain activities (Chavez et al., 2010). Furthermore, small-worldness has been reported to be characteristic of a healthy brainbecause lower levels signify abnormal brain functioning (Brier et al., 2014; Liu et al., 2008). Moreover, strong chronotype preference was positively correlated with path length and assortativity in the evening session. Higher assortativity is another sign of a well-functioning network, confirming that individuals with a stronger chronotype preference present higher integration of the neuronal networks in the evenings compared to the mornings. As a result, the more extreme the chronotype, the more integrated the neuronal network in the evening, which was a finding that was not dependent on the chronotype itself. Sleepiness during the day was positively correlated with the average path length and assortativity during the morning hours.

Local graph measures revealed significant differences in resting-state activity associated with the preferred chronotype. People with the ET chronotype revealed less centrality in the somatomotor and limbic networks during the morning hours, which is believed to be associated with less efficiency. These results also confirm previous findings concerning the effect of sleep inertia on later chronotypes (Ritchie et al., 2017).

Furthermore, declared sleepiness during the day negatively correlated with the centrality of the DMN structure during the morning session. Interestingly, according to Tian et al. (2020), the DMN mediates the association between chronotype and sleep quality. The same study displayed higher precuneus and medial prefrontal cortex connectivity in late chronotypes. Moreover, during the evening session, the strength of the chronotype preferences positively correlated with the degree of centrality in several regions within the visual network, such as the middle and superior occipital gyrus and fusiform gyrus. These regions are believed to be associated with visual attention.

### 4.6. Limitations and future directions

Certain limitations associated with this study should be considered in future research. Firstly, the relatively small number of subjects in this study might constrain the translational value of our results. Future studies with larger samples are needed to confirm our findings and increase the reproducibility of the research.

Secondly, brain nodes were derived from the cortical Schaefer-Yeo atlas (200-parcel/7-network parcellation; Schaefer et al., 2018). Schaefer’s parcellations are available at multiple resolutions (100 to 1000 parcels). Further studies on appraising network topology using finer parcellation schemes are warranted. Also, because the Schaefer atlas considers only cortical areas, whole-brain studies could be carried out by adding subcortical regions with other atlases or segmentation algorithms. Although each node in this atlas is preassigned to a system/network (which is an advantage over many atlases), the Schaefer system labels do not allow for individual variation in the topography of brain systems; that is, they force everyone to have the same systems, which may not be appropriate and it is an issue that requires more attention (Gordon et al., 2017; Kong et al., 2019).

A third limitation concerns the measures used to compare the group-representative functional brain networks with one another. These measures tend to be correlated with one another; for example, a brain network with high efficiency must necessarily have a shorter path length (Betzel et al., 2018). Thus, if we find significant differences in one measure, they will probably be found in others. Our analyses could therefore be extended in future work to determine which of these measures might be driving the others and how an exhaustive set of metrics might be designed to fit the study specification from a neurobiological perspective. Another issue about network measures is that global statistics are often non-specific (i.e., they are not entirely informative and revealing). For example, the meaning of the phrase “the patient group has a lower efficiency than the control” may not be apparent to a neurosurgeon. Future work should be directed to better interpretation of such metrics.

Another limitation concerns the applicability of the small-world property. In real systems, the early definitions of small-worldness initiated by Watts and Strogatz (1998) are ineffective because they confuse regular networks with small-world structures and neglect the weight and physical length of connections and the network density. Most of the definitions present the network on the border of a circle; however, real systems are not embedded in this way. There are many ways for a network to be small-world other than starting from a regular grid and adding random links to reduce path length. Numerous researchers have addressed these constraints by introducing several practical metrics (Bolaños et al., 2013; Muldoon et al., 2016; Rubinov and Sporns, 2010; Telesford et al., 2011). Applying these modified metrics in future work would bring the study of the small-world brain closer to reality.

Yet another possible extension of this work involves studying the recent theory-driven techniques that emphasize the importance of machine learning, algorithmic optimization, and parallel computing in functional neuroimaging (Cohen et al., 2017; Douglas et al., 2013). For example, various algorithms known as graph neural networks, including graph convolutional networks (GCNs), have been proposed to show how graph theory can be used to train deep learning models (Kipf and Welling, 2016; Wu et al., 2020) and to discover neurological biomarkers using fMRI data (Li et al., 2020). As another example, a growing trend has developed in a family of algorithms known as hyperalignment (or functional alignment) that permit a projection of individuals’ data into a shared space across participants based on how voxels respond to stimuli (Guntupalli et al., 2016; Haxby et al., 2011) or how they are connected to other voxels (Guntupalli et al., 2018; Haxby et al., 2020). Combining these techniques with network neuroscience will open a new generation of studies to transform our knowledge of neural representations in complex brain networks.

## 5. Conclusion

This study, presents evidence for topological changes in functional brain networks throughout the day (TOD effect; morning and evening sessions) using rs-fMRI data and graph theory analysis. We also consider inter-individual differences in diurnal preferences (chronotype effect; “lark” or “owl” types) in addition to the impact of TOD. In summary, the results from the global examination showed more efficient functional topology in the evening session, regardless of the chronotype. Moreover, the mesoscale results represented how different systems/regions interacted with one another at both sessions, providing an intuitive assessment of modular organization using measures of recruitment and integration. To counterbalance, the local analysis revealed chronotype-specific modulation of diurnal fluctuation prominently across the somatomotor, ventral attention, and default-mode networks. These findings provide insight into diurnal variations in resting-brain networks, reflecting the universal effect of TOD on neural functional architecture when designing experiments. The findings also indicate the need to control for circadian typology, which could influence experimental results in neuroimaging studies.

## Figures and Tables

**Table 1:** Summary statistics for demographics and questionnaires.

Variables (mean ± SD)	MT (*n* = 31)	ET (*n* = 31)	Significance
Sex (M/F)^a^	11/20	12/19	X^2^(1) = 0.069; *p* = 0.793
Age (years)^b^	24.45 ± 3.83	23.48 ± 2.55	U(62) = 446; *p* = 0.623
Declared wake-up time (hh:mm)^c^	07:07 ± 62 min	07:25 ± 48 min	t(60) = −1.9; *p* = 0.062
Declared bedtime (hh:mm)^c^	23:24 ± 55 min	00:06 ± 49 min	t(60) = −3.5; *p* = 0.001
Declared amount of perfect sleep (hh:mm)^c^	08:50 ± 42 min	08:38 ± 54 min	t(60) = 1.54; *p* = 0.128
ME^b^	15.71 ± 2.41	28.45 ± 3.83	U(62) < 0.001; *p* = 0.623
AM^b^	21.47 ± 3.58	22.26 ± 3.51	U(62) = 426; *p* = 0.437
ESS^b^	5.52 ± 2.48	5.87 ± 3.01	U(62) = 441; *p* = 0.576
EHI^b^	86.83 ± 12.92	89.19 ± 13.93	U(62) = 414; *p* = 0.330
VNTR of PER3	5/5	4/4	–

MT – morning types, ET – evening types, ME – morningness/eveningness scale (Chronotype Questionnaire), AM – amplitude scale (Chronotype Questionnaire), ESS – Epworth Sleepiness Scale, EHI – Epworth Handedness Inventory,.

a chi-square test,.

b Mann-Whitney U Test,.

c Student’s *t*-test.

**Table 2:** Summary statistics for actigraphy.

Variables (mean ± SD)	MT (*n* = 31)	ET (*n* = 31)	Significance
Actigraphy-derived wake-up time (hh:mm)^c^	7:43 ± 70 min	8:16 ± 69 min	t(60) = −1.28; *p* = 0.168
Actigraphy-derived bedtime (hh:mm)^c^	23:58 ± 58 min	0:48 ± 58 min	t(60) = −3.13; *p* = 0.002
Actigraphy-derived length of real sleep (hh:mm)^c^	7:53.83 ± 51 min	7:36 ± 40 min	t(60) = −1.18; *p* = 0.266

MT – morning types, ET – evening types,.

c Student’s *t*-test.

**Table 3:** Mathematical definition and explanation of the measures used in this study.

Description	Equation
**Local measures**	
*Degree*: the number of neighbors (connections) for each node	For a given node *i*: $k_{i}=\sum_{j\in N}a_{ij}$ *N*: set of all nodes in the network and *a_ij_*: connection between *i* and *j*: *a_ij_* = 1 when the link (*i, j*) exists, and 0 otherwise. There are no self-loops in the network; therefore *a_ii_* = 0.
*Path length*: the potential for information transmission along the shortest paths, calculated as the average distance from one node to all other nodes	Path length for a given node *i* (Watts and Strogatz, 1998): $L_{i}=\frac{\sum_{j\in N,j\neq i}d_{ij}}{n-1}$ *n*: Number of nodes and *d_ij_*: shortest path length (distance) between nodes *i* and *j*.
*Clustering coefficient*: the extent to which the neighbors of a given node are interconnected (i.e., the fraction of triangles around a node)	Clustering coefficient of the network (Watts and Strogatz, 1998): $C_{i}=\frac{\sum_{j,k\in N}a_{ij}a_{ik}a_{jk}}{k_{i}\left(k_{i}-1\right)}$
*Efficiency*: the efficiency of parallel information transfer of a given node determined as the average of the reciprocal shortest path length from a node to all other nodes	Efficiency for a given node *i* (Latora and Marchiori, 2001): $E_{i}=\frac{\sum_{j\in N,j\neq i}d_{ij}^{-1}}{n-1}$
*Betweenness centrality*: the ratio of all shortest paths in the graph that contain a given node	Betweenness centrality of node *i* (Freeman, 1978): $\begin{array}{l} \begin{array}{ccc} BC_{i}=\frac{1}{\left(n-1\right)\left(n-2\right)} & \Sigma & \frac{\rho_{hj}\left(i\right)}{\rho_{hj}} \end{array}\\ h,j\in N\\ h\neq j,h\neq i,j\neq i \end{array}$ *ρ_hj_*: number of shortest paths between *h* and *j* and *ρ_hj_*(*i*): number of shortest paths between *h* and *j* that use *i*.
*Participation coefficient*: the distribution of a node’s connections across its modules	Participation coefficient of node *i* (Guimerà and Nunes Amaral, 2005): $P_{i}=1-\sum_{m\in M}{\left(\frac{k_{i}\left(m\right)}{k_{i}}\right)}^{2}$ *M*: set of non-overlapping modules and *k_i_*(*m*): number of links between *i* and all nodes in module *m*.
**Global measures**	
*Characteristic path length*: average path lengths over all nodes	Characteristic path length of the network (Watts and Strogatz, 1998): $L=\frac{1}{n}\sum_{i\in N}L_{i}$ *L_i_*: average distance from node *i* to all other nodes (path length).
*Clustering coefficient*: average of the nodal clustering coefficients over all nodes	Clustering coefficient of the network (Watts and Strogatz, 1998): $C=\frac{1}{n}\sum_{i\in N}C_{i}$ *C_i_*: clustering coefficient of node *i*.
*Small-worldness*: an assessment used for networks in which most nodes are not adjacent to each other, but can be accessed by any other node with the minimum possible path length. Small-world networks are considered an intermediate between regular and random networks (i.e., they contain many short-range connections alongside a few long-range links), indicating a high clustering coefficient and a short path length.	Small-worldness of the network (Mark D. Humphries and Gurney, 2008): $\sigma =\frac{C_{net}/C_{rand}}{L_{net}/L_{rand}}$ *C_net_* and *L_net_* are clustering coefficient and path length of a given network, and *C_rand_* and *L_rand_* are these measures for an equivalent random network. Small-world networks have *σ* ⪢1.
*Efficiency*: average of the nodal efficiencies over all nodes	Global efficiency of the network (Latora and Marchiori, 2001): $E=\frac{1}{n}\sum_{i\in N}E_{i}$ *E_i_*: efficiency of node *i*.
*Assortativity*: the extent to which a network can resist failures in its main components. If *r* ≥ 0, the nodes with a high degree are more likely to connect to others that are similar in degree (an assortative network), while *r* < 0 reflects a tendency for high-degree nodes to attach to nodes with a low degree (a disassortative network).	Assortativity coefficient of the network (Newman, 2002): $r=\frac{\frac{1}{I}\sum_{\left(i,j\right)\in L}k_{i}k_{j}-\left[\frac{1}{I}\sum_{\left(i,j\right)\in L}\frac{1}{2}{\left(k_{i}+k_{j}\right)}^{2}\right]}{\frac{1}{I}\sum_{\left(i,j\right)\in L}\frac{1}{2}\left(k_{i}^{2}+k_{j}^{2}\right)-\left[\frac{1}{I}\sum_{\left(i,j\right)\in L}\frac{1}{2}{\left(k_{i}+k_{j}\right)}^{2}\right]}$
*Synchronization*: an examination of how network nodes fluctuate in the same wave pattern	Synchronization of the network (Barahona and Pecora, 2002): $S=\frac{\lambda \left(2\right)}{\lambda \left(M\right)}$ *λ*(2): second smallest eigenvalue of the matrix of *A*, *λ*(*M*): largest eigenvalue of the matrix of *A*, and *A*: adjacency matrix of the network.
**Mesoscale measures**	
*Modularity (single-slice)*: it reflects the quality of partitioning a network into clusters of densely interconnected nodes with sparse connections among other clusters	Single-slice modularity of the network (Newman, 2004): $Q_{singleslice}=\frac{1}{2\mu }\sum_{ij}\left[A_{ij}-\gamma V_{ij}\right]\delta \left(\sigma_{i},\sigma_{j}\right)$ *A_ij_* and *V_ij_*: observed and expected weights of the connection between nodes *i* and *j* *μ*: total edge weight in the network, *γ*: structural resolution parameter, *σ_i_*: community (i.e., “module”) assignment of node *i*, and *δ*(*x*, *y*): Kronecker delta function; it takes on a value of 1 when *x* = *y*, and 0 otherwise (assuming that the given network consists of *M* non-overlapping modules).
*Modularity (multi-slice)*: in the multi-layer version of the modularity function, nodes are connected to themselves across layers by an inter-layer coupling parameter, *ω*	Multi-slice modularity of the network (Mucha et al., 2010): $Q_{multislice}=\frac{1}{2\mu }\sum_{ijsr}\left[\left(A_{ijs}-\gamma V_{ijs}\right)\delta_{sr}+\omega \delta_{ij}\right]\delta \left(\sigma_{is},\sigma_{jr}\right)$ *A_ijs_* and *V_ijs_*: observed and expected weight of the connection between nodes *i* and *j* in layer *s*, *μ*, *γ*, and *δ*(*x, y*): defined as above, *ω*: interlayer coupling parameter, *σ_is_*: community assignment of node *i* in slice *s*, and *σ_jr_*: community assignment of node *j* in slice *r*.
*Recruitment*: the fraction of layers in which a node is assigned to the same community as other nodes from the same predefined/static system	Recruitment of node (region) *i* in system *S* (Bassett et al., 2015): $R_{i}^{S}=\frac{1}{n_{s}}\sum_{j\in S}P_{ij}$ *n_s_*: number of regions in *S* and *P_ij_*: module allegiance between node *i* and node *j*. Module allegiance represents the fraction of layers in which nodes i and j are assigned to the same community. To construct an allegiance matrix, a co-occurrence matrix (*N* × *N*) for each layer is created, wherein the *ij^th^* element is equal to 1 if the nodes *i* and *j* have a shared community label, and 0 otherwise. The average of all co-occurrence matrices across layers forms the allegiance matrix, so its elements range from 0 to 1.
*Integration*: the fraction of layers in which a given node in system S is assigned to the same community as nodes from systems other than S	Integration of node *i* in system *S* (Bassett et al., 2015): $I_{i}^{S}=\frac{1}{N-n_{s}}\sum_{j\notin S}P_{ij}$ N: total number of nodes (regions).

**Table 4:** List of brain regions of interest (ROIs) that differed significantly between morning and evening sessions (t: time) and between the “lark” and “owl” participants (c: chronotype). P-values were computed using 30,000 permutations followed by Benjamini–Hochberg correction in a two-way ANOVA; FDR was set to 0.05 and the asterisks indicate statistically significant corrected p-values).

ROI	Schaefer node label	Cortical areas	MNI coordinates			Adjusted *p*-value			
			x	y	z	Degree Centrality	Betweenness Centrality	Clustering Coefficient	Nodal Efficiency
15	LH_SomMot_1	LH_Superior temporal gyrus	−51	−4	−2	* (t)			* (t)
30	LH_SomMot_16	LH_Postcentral gyrus	−19	−31	68	* (t)			* (t)
42	LH_DorsAttn_FEF_2	LH_Superior frontal gyrus (posterior segment)	−22	6	62			* (c)	* (c)
46	LH_SalVentAttn_ParOper_3	LH_Supramarginal gyrus	−60	−39	36	* (t)			
48	LH_SalVentAttn_FrOperIns_2	LH_Insular	−33	20	5	* (c)			* (c)
51	LH_SalVentAttn_PFCl_1	LH_Middle frontal gyrus (dorsal prefrontal cortex)	−28	43	31	* (t)			* (t)
63	LH_Cont_Par_3	LH_Angular gyrus	−45	−42	46				* (t)
79	LH_Default_Par_1	LH_Posterior middle temporal gyrus	−48	−57	18		* (c)		
90	LH_Default_PFC_8	LH_Dorsal anterior cingulate gyrus	−6	30	25	* (c)	* (c)		* (c)
94	LH_Default_PFC_12	LH_Superior frontal gyrus (posterior segment)	−24	25	49			* (t)	
99	LH_Default_pCunPCC_4	LH_Precuneous	−6	−54	42		* (t)		
117	RH_SomMot_2	RH_Superior temporal gyrus	64	−23	8	* (t)		* (t)	
126	RH_SomMot_11	RH_Precentral gyrus	7	−11	51		* (c)		
138	RH_DorsAttn_Post_4	RH_Angular gyrus	46	−38	49		* (t)	* (t)	
145	RH_DorsAttn_FEF_1	RH_Precentral gyrus	34	−4	52		* (c)		
150	RH_SalVentAttn_TempOccPar_3	RH_Supramarginal gyrus	60	−26	27		* (t)		
180	RH_Cont_PFCmp_1	RH_Dorsal anterior cingulate gyrus	7	31	28	* (c)	* (c)		* (c)
187	RH_Default_Temp_3	RH_Superior temporal gyrus	55	−6	−10			* (t)	
200	RH_Default_pCunPCC_3	RH_Precuneous	6	−58	44		* (t)		

Abbreviations: *MNI* – Montreal Neurological Institute space; *LH* – left hemisphere; *RH* – right hemisphere; *t* – time; *c* – chronotype; *SomMot*_[*i*] – *i*th segment of the Somatomotor Network; *DorsAttn_Post_4* – fourth segment of the posterior Dorsal Attentional Network; *DorsAttn_FEF_[i]* – *i*th segment of the frontal eye fields Dorsal Attentional Network; *SalVentAttn_ParOper_3* – third segment of the parietal operculum Salience/Ventral Attention Network; *SalVentAttn_TempOccPar_3* – third segment of the temporal occipital parietal Salience/Ventral Attention Network; *SalVentAttn_FrOperIns_2* – second segment of the frontal operculum insula Salience/Ventral Attention Network; *SalVentAttn_PFCl_1* – first segment of the lateral prefrontal cortex Salience/Ventral Attention Network; *Cont_Par_3* – third segment of the parietal Control Network; *Cont_PFCmp_1* – first segment of the medial posterior prefrontal cortex Control Network; *Default_Temp_3* – third segment of the temporal Default Network; *Default_ Par_1* – first segment of the parietal Default Network; *Default_PFC_[i]* – *i*th segment of the prefrontal cortex Default Network; *Default_ pCunPCC_[i]* – *i*th segment of the precuneus posterior cingulate cortex Default Network.

Labels from the Yeo and Schaefer Atlas are available here.

**Table 5:** Global analysis: significant correlations between global metrics and AM, ESS, and ME scores (*n* = 62) for the morning and evening sessions. The significance level was set at *p* < 0.05 with non-parametric permutations.

	Correlation (adjusted *p*-value)					
	Morning session			Evening session		
	AM	ESS	ME	AM	ESS	ME
Small-worldness	−0.44 (0.0003)	—	—	—	—	—
Path length	—	0.30 (0.0183)	—	0.28 (0.0224)	—	—
Modularity	−0.46 (0.0002)	—	—	—	—	—
Assortativity	—	0.29 (0.0230)	—	0.29 (0.0225)	—	—

Abbreviations: *ME* – morningness/eveningness scale; *AM* – amplitude scale; *ESS* – Epworth Sleepiness Scale.

Information about the Yeo and Schaefer atlas can be accessed from here.

**Table 6:** Nodal analysis: significant correlations between degree centrality and AM, ESS, and ME scores (*n* = 62) for the morning and evening sessions. The significance level was set at *p* < 0.05 with non-parametric permutations.

ROI (Schaefer-Yeo Atlas)			Correlation (adjusted *p*-value)					
			Morning Session			Evening Session		
			AM	ESS	ME	AM	ESS	ME
Left Hemisphere	11	Vis_11				0.30 (0.0189)	0.37 (0.0024)	0.37 (0.0024)
	18	SomMot_4			−0.37 (0.0029)			
	41	DorsAttn_FEF_1	0.38 (0.0023)					
	43	DorsAttn_PrCv_1				−0.33 (0.0091)		
	48	SalVentAttn_FrOper_2			0.37 (0.0024)			
	55	Limbic_OFC_1				−0.32 (0.0149)		
	59	Limbic_TempPole_3		−0.42 (0.0007)				
	60	Limbic_TempPole_4			−0.33 (0.0076)			
	88	Default_PFC_6	−0.32 (0.0113)					
	90	Default_PFC_8			0.30 (0.0200)			
	97	Default_pCunPCC_2	−0.39 (0.0015)					
	99	Default_ pCunPCC_4	−0.32 (0.0093)					
Right Hemisphere	102	Vis_2				0.30 (0.0191)		
	114	Vis_14				0.31 (0.0159)		
	126	SomMot_11	0.30 (0.0172)					
	145	DorsAttn_FEF_1			0.33 (0.0080)			
	160	Limbic_OFC_2				−0.32 (0.0116)		
	169	Cont_PFCv_1			0.31 (0.0134)			
	170	Cont_PFCl_1		−0.31 (0.0153)				
	185	Default_Temp_1		−0.30 (0.0177)				
	188	Default_Temp_4				−0.31 (0.0128)		
	194	Default_PFCm_4	−0.37 (0.0032)					
	199	Default_pCunPCC_2	−0.29 (0.0195)					

Abbreviations: *MT* – morning type; *ET* – evening type; *ME* – morningness/eveningness scale; *AM* – amplitude scale; *ESS* – Epworth Sleepiness Scale.

Information about the Yeo and Schaefer atlas can be accessed here.

## Appendix

Table A1

**Table A1:** Summary of Shaefer/Yeo parcellation label names, component names, corresponding Montreal Neurological Institute (MNI) coordinates, and the associated RGB codes used in the connectograms (7 networks, 200 nodes).

	Label name	Component name	MNI coordinates	RGB code
Visual Network (VN), Left Hemisphere				
1	LH_Vis_1	Visual	−24,−53,−9	123,104,238
2	LH_Vis_2	Visual	−26,−77,−14	147,112,219
3	LH_Vis_3	Visual	−45,−69,−8	138,43,226
4	LH_Vis_4	Visual	−10,−67,−4	201,160,220
5	LH_Vis_5	Visual	−27,−95,−12	204,204,255
6	LH_Vis_6	Visual	−14,−44,−3	75,0,130
7	LH_Vis_7	Visual	−5,−93,−4	181,126,220
8	LH_Vis_8	Visual	−47,−70,10	147,112,219
9	LH_Vis_9	Visual	−23,−97,6	167,107,207
10	LH_Vis_10	Visual	−11,−70,7	116,108,192
11	LH_Vis_11	Visual	−40,−85,11	138,43,226
12	LH_Vis_12	Visual	−12,−73,22	111,0,255
13	LH_Vis_13	Visual	−7,−87,28	120,81,169
14	LH_Vis_14	Visual	−23,−87,23	115,79,150
Somatomotor Network (SMN), Left Hemisphere				
15	LH_SomMot_1	Somatomotor	−51,−4,−2	135,206,235
16	LH_SomMot_2	Somatomotor	−53,−24,9	30,144,255
17	LH_SomMot_3	Somatomotor	−37,−21,16	0,191,255
18	LH_SomMot_4	Somatomotor	−55,−4,10	0,0,205
19	LH_SomMot_5	Somatomotor	−53,−22,18	172,229,238
20	LH_SomMot_6	Somatomotor	−56,−8,31	135,206,250
21	LH_SomMot_7	Somatomotor	−47,−9,46	119,181,254
22	LH_SomMot_8	Somatomotor	−7,−12,46	79,134,247
23	LH_SomMot_9	Somatomotor	−49,−28,57	119,158,203
24	LH_SomMot_10	Somatomotor	−40,−25,57	65,102,245
25	LH_SomMot_11	Somatomotor	−31,−46,63	69,177,232
26	LH_SomMot_12	Somatomotor	−32,−22,64	49,140,231
27	LH_SomMot_13	Somatomotor	−26,−38,68	73,151,208
28	LH_SomMot_14	Somatomotor	−20,−11,68	15,192,252
29	LH_SomMot_15	Somatomotor	−5,−29,67	65,125,193
30	LH_SomMot_16	Somatomotor	−19,−31,68	0,127,255
Dorsal Attention Network (DAN), Left Hemisphere				
31	LH_DorsAttn_Post_1	Posterior	−43,−48,−19	0,255,127
32	LH_DorsAttn_Post_2	Posterior	−57,−60,−1	50,205,50
33	LH_DorsAttn_Post_3	Posterior	−26,−70,38	173,255,47
34	LH_DorsAttn_Post_4	Posterior	−54,−27,42	144,238,144
35	LH_DorsAttn_Post_5	Posterior	−41,−35,47	60,179,113
36	LH_DorsAttn_Post_6	Posterior	−33,−49,47	34,139,34
37	LH_DorsAttn_Post_7	Posterior	−17,−73,54	152,255,152
38	LH_DorsAttn_Post_8	Posterior	−29,−60,59	144,238,144
39	LH_DorsAttn_Post_9	Posterior	−6,−60,57	119,221,119
40	LH_DorsAttn_Post_10	Posterior	−17,−53,68	116,195,101
41	LH_DorsAttn_FEF_1	Frontal Eye Fields	−31,−4,53	80,200,120
42	LH_DorsAttn_FEF_2	Frontal Eye Fields	−22,6,62	57,255,20
43	LH_DorsAttn_PrCv_1	Precentral Ventral	−48,6,29	34,139,34
Ventral Attention Network (DAN), Left Hemisphere				
44	LH_SalVentAttn_ParOper_1	Parietal Operculum	−56,−40,20	249,132,229
45	LH_SalVentAttn_ParOper_2	Parietal Operculum	−61,−26,28	254,78,218
46	LH_SalVentAttn_ParOper_3	Parietal Operculum	−60,−39,36	207,113,175
47	LH_SalVentAttn_FrOperIns_1	Frontal Operculum Insula	−39,−4,−4	189,51,164
48	LH_SalVentAttn_FrOperIns_2	Frontal Operculum Insula	−33,20,5	204,0,204
49	LH_SalVentAttn_FrOperIns_3	Frontal Operculum Insula	−39,1,11	218,112,214
50	LH_SalVentAttn_FrOperIns_4	Frontal Operculum Insula	−51,9,11	241,167,254
51	LH_SalVentAttn_PFCl_1	Lateral Prefrontal Cortex	−28,43,31	238,130,238
52	LH_SalVentAttn_Med_1	Medial	−6,9,41	255,111,255
53	LH_SalVentAttn_Med_2	Medial	−11,−35,46	207,52,118
54	LH_SalVentAttn_Med_3	Medial	−6,−3,65	223,0,255
Limbic Network (LN), Left Hemisphere				
55	LH_Limbic_OFC_1	Orbital Frontal Cortex	−24,22,−20	255,248,220
56	LH_Limbic_OFC_2	Orbital Frontal Cortex	−10,35,−21	240,230,140
57	LH_Limbic_TempPole_1	Temporal Pole	−29,−6,−39	252,247,94
58	LH_Limbic_TempPole_2	Temporal Pole	−45,−20,−30	255,250,205
59	LH_Limbic_TempPole_3	Temporal Pole	−28,10,−34	251,236,93
60	LH_Limbic_TempPole_4	Temporal Pole	−43,8,−19	255,247,0
Frontoparietal Network (FPN), Left Hemisphere				
61	LH_Cont_Par_1	Parietal	−53,−51,46	255,165,0
62	LH_Cont_Par_2	Parietal	−35,−62,48	255,140,0
63	LH_Cont_Par_3	Parietal	−45,−42,46	255,160,137
64	LH_Cont_Temp_1	Temporal	−61,−43,−13	255,200,124
65	LH_Cont_OFC_1	Orbital Frontal Cortex	−32,42,−13	255,153,102
66	LH_Cont_PFCl_1	Lateral Prefrontal Cortex	−42,49,−6	255,163,67
67	LH_Cont_PFCl_2	Lateral Prefrontal Cortex	−28,58,8	255,130,67
68	LH_Cont_PFCl_3	Lateral Prefrontal Cortex	−42,40,16	255,174,66
69	LH_Cont_PFCl_4	Lateral Prefrontal Cortex	−44,20,27	237,135,45
70	LH_Cont_PFCl_5	Lateral Prefrontal Cortex	−43,6,43	224,141,60
71	LH_Cont_pCun_1	Precuneus	−9,−73,38	255,153,51
72	LH_Cont_Cing_1	Cingulate	−5,−29,28	237,145,33
73	LH_Cont_Cing_2	Cingulate	−3,4,30	251,153,2
Default Mode Network (DMN), Left Hemisphere				
74	LH_Default_Temp_1	Temporal	−47,8,−33	240,128,128
75	LH_Default_Temp_2	Temporal	−60,−19,−22	255,69,0
76	LH_Default_Temp_3	Temporal	−56,−6,−12	165,42,42
77	LH_Default_Temp_4	Temporal	−58,−30,−4	255,0,0
78	LH_Default_Temp_5	Temporal	−58,−43,7	123,17,19
79	LH_Default_Par_1	Parietal	−48,−57,18	204,51,51
80	LH_Default_Par_2	Parietal	−39,−80,31	205,92,92
81	LH_Default_Par_3	Parietal	−57,−54,28	253,94,83
82	LH_Default_Par_4	Parietal	−46,−66,38	127,23,52
83	LH_Default_PFC_1	Prefrontal Cortex	−35,20,−13	255,53,94
84	LH_Default_PFC_2	Prefrontal Cortex	−6,36,−10	235,76,66
85	LH_Default_PFC_3	Prefrontal Cortex	−46,31,−7	204,78,92
86	LH_Default_PFC_4	Prefrontal Cortex	−12,63,−6	178,34,34
87	LH_Default_PFC_5	Prefrontal Cortex	−52,22,8	203,65,84
88	LH_Default_PFC_6	Prefrontal Cortex	−6,44,7	237,28,36
89	LH_Default_PFC_7	Prefrontal Cortex	−8,59,21	218,44,67
90	LH_Default_PFC_8	Prefrontal Cortex	−6,30,25	229,26,76
91	LH_Default_PFC_9	Prefrontal Cortex	−11,47,45	255,36,0
92	LH_Default_PFC_10	Prefrontal Cortex	−3,33,43	255,69,0
93	LH_Default_PFC_11	Prefrontal Cortex	−40,19,49	171,75,82
94	LH_Default_PFC_12	Prefrontal Cortex	−24,25,49	156,37,66
95	LH_Default_PFC_13	Prefrontal Cortex	−9,17,63	194,59,34
96	LH_Default_pCunPCC_1	Precuneus/Posterior Cingulate Cortex	−11,−56,13	196,30,58
97	LH_Default_pCunPCC_2	Precuneus/Posterior Cingulate Cortex	−5,−55,27	255,64,64
98	LH_Default_pCunPCC_3	Precuneus/Posterior Cingulate Cortex	−4,−31,36	211,0,63
99	LH_Default_pCunPCC_4	Precuneus/Posterior Cingulate Cortex	−6,−54,42	157,41,51
100	LH_Default_PHC_1	Parahippocampal Cortex	−26,−32,−18	205,92,92
Visual Network (VN), Right Hemisphere				
101	RH_Vis_1	Visual	39,−35,−23	123,104,238
102	RH_Vis_2	Visual	28,−36,−14	147,112,219
103	RH_Vis_3	Visual	29,−69,−12	138,43,226
104	RH_Vis_4	Visual	12,−65,−5	201,160,220
105	RH_Vis_5	Visual	48,−71,−6	204,204,255
106	RH_Vis_6	Visual	11,−92,−5	75,0,130
107	RH_Vis_7	Visual	16,−46,−1	181,126,220
108	RH_Vis_8	Visual	31,−94,−4	147,112,219
109	RH_Vis_9	Visual	9,−75,9	167,107,207
110	RH_Vis_10	Visual	22,−60,7	116,108,192
111	RH_Vis_11	Visual	42,−80,10	138,43,226
112	RH_Vis_12	Visual	20,−90,22	111,0,255
113	RH_Vis_13	Visual	11,−74,26	120,81,169
114	RH_Vis_14	Visual	16,−85,39	115,79,150
115	RH_Vis_15	Visual	33,−75,32	105,53,156
Somatomotor Network (SMN), Right Hemisphere				
116	RH_SomMot_1	Somatomotor	51,−15,5	135,206,235
117	RH_SomMot_2	Somatomotor	64,−23,8	30,144,255
118	RH_SomMot_3	Somatomotor	38,−13,15	0,191,255
119	RH_SomMot_4	Somatomotor	44,−27,18	0,0,205
120	RH_SomMot_5	Somatomotor	59,0,10	172,229,238
121	RH_SomMot_6	Somatomotor	56,−11,14	135,206,250
122	RH_SomMot_7	Somatomotor	58,−5,31	119,181,254
123	RH_SomMot_8	Somatomotor	10,−15,41	79,134,247
124	RH_SomMot_9	Somatomotor	51,−22,52	119,158,203
125	RH_SomMot_10	Somatomotor	47,−11,48	65,102,245
126	RH_SomMot_11	Somatomotor	7,−11,51	69,177,232
127	RH_SomMot_12	Somatomotor	40,−24,57	49,140,231
128	RH_SomMot_13	Somatomotor	32,−40,64	73,151,208
129	RH_SomMot_14	Somatomotor	33,−21,65	15,192,252
130	RH_SomMot_15	Somatomotor	29,−34,65	65,125,193
131	RH_SomMot_16	Somatomotor	22,−9,67	0,127,255
132	RH_SomMot_17	Somatomotor	10,−39,69	0,191,255
133	RH_SomMot_18	Somatomotor	6,−23,69	29,172,214
134	RH_SomMot_19	Somatomotor	20,−29,70	25,116,210
Dorsal Attention Network (DAN), Right Hemisphere				
135	RH_DorsAttn_Post_1	Posterior	50,−53,−15	0,255,127
136	RH_DorsAttn_Post_2	Posterior	52,−60,9	50,205,50
137	RH_DorsAttn_Post_3	Posterior	59,−16,34	173,255,47
138	RH_DorsAttn_Post_4	Posterior	46,−38,49	144,238,144
139	RH_DorsAttn_Post_5	Posterior	41,−31,46	60,179,113
140	RH_DorsAttn_Post_6	Posterior	15,−73,53	34,139,34
141	RH_DorsAttn_Post_7	Posterior	34,−48,51	152,255,152
142	RH_DorsAttn_Post_8	Posterior	26,−61,58	144,238,144
143	RH_DorsAttn_Post_9	Posterior	8,−56,61	119,221,119
144	RH_DorsAttn_Post_10	Posterior	21,−48,70	116,195,101
145	RH_DorsAttn_FEF_1	Frontal Eye Fields	34,−4,52	80,200,120
146	RH_DorsAttn_FEF_2	Frontal Eye Fields	26,7,58	57,255,20
147	RH_DorsAttn_PrCv_1	Precentral Ventral	52,11,21	34,139,34
Ventral Attention Network (VAN), Right Hemisphere				
148	RH_SalVentAttn_TempOccPar_1	Temporal Occipital Parietal	57,−45,9	255,0,144
149	RH_SalVentAttn_TempOccParr_2	Temporal Occipital Parietal	60,−39,17	218,29,129
150	RH_SalVentAttn_TempOccParr_3	Temporal Occipital Parietal	60,−26,27	255,111,255
151	RH_SalVentAttn_PrC_1	Precentral	51,4,40	189,51,164
152	RH_SalVentAttn_FrOperIns_1	Frontal Operculum Insula	41,6,−15	189,51,164
153	RH_SalVentAttn_FrOperIns_2	Frontal Operculum Insula	46,−4,−4	204,0,204
154	RH_SalVentAttn_FrOperIns_3	Frontal Operculum Insula	36,24,5	218,112,214
155	RH_SalVentAttn_FrOperIns_4	Frontal Operculum Insula	43,7,4	241,167,254
156	RH_SalVentAttn_Med_1	Medial	7,9,41	255,111,255
157	RH_SalVentAttn_Med_2	Medial	11,−36,47	207,52,118
158	RH_SalVentAttn_Med_3	Medial	8,3,66	223,0,255
Limbic Network (LN), Right Hemisphere				
159	RH_Limbic_OFC_1	Orbital Frontal Cortex	12,39,−22	255,248,220
160	RH_Limbic_OFC_2	Orbital Frontal Cortex	28,22,−19	240,230,140
161	RH_Limbic_OFC_3	Orbital Frontal Cortex	15,64,−8	253,253,150
162	RH_Limbic_TempPole_1	Temporal Pole	30,9,−38	252,247,94
163	RH_Limbic_TempPole_2	Temporal Pole	47,−12,−35	255,250,205
164	RH_Limbic_TempPole_3	Temporal Pole	25,−11,−32	251,236,93
Frontoparietal Network (FPN), Right Hemisphere				
165	RH_Cont_Par_1	Parietal	62,−37,37	255,165,0
166	RH_Cont_Par_2	Parietal	53,−42,48	255,140,0
167	RH_Cont_Par_3	Parietal	37,−63,47	255,160,137
168	RH_Cont_Temp_1	Temporal	63,−41,−12	255,200,124
169	RH_Cont_PFCv_1	Ventral Prefrontal Cortex	34,21,−8	255,103,0
170	RH_Cont_PFCl_1	Lateral Prefrontal Cortex	36,46,−13	255,153,102
171	RH_Cont_PFCl_2	Lateral Prefrontal Cortex	29,58,5	255,163,67
172	RH_Cont_PFCl_3	Lateral Prefrontal Cortex	43,45,10	255,130,67
173	RH_Cont_PFCl_4	Lateral Prefrontal Cortex	46,24,26	255,174,66
174	RH_Cont_PFCl_5	Lateral Prefrontal Cortex	30,48,27	237,135,45
175	RH_Cont_PFCl_6	Lateral Prefrontal Cortex	41,33,37	224,141,60
176	RH_Cont_PFCl_7	Lateral Prefrontal Cortex	42,14,49	255,167,0
177	RH_Cont_pCun_1	Precuneus	14,−70,37	255,153,51
178	RH_Cont_Cing_1	Cingulate	5,−24,31	237,145,33
179	RH_Cont_Cing_2	Cingulate	5,3,30	251,153,2
180	RH_Cont_PFCmp_1	Medial Posterior Prefrontal Cortex	7,31,28	255,186,0
181	RH_Cont_PFCmp_2	Medial Posterior Prefrontal Cortex	7,25,55	228,155,15
Default Mode Network (DMN), Right Hemisphere				
182	RH_Default_Par_1	Parietal	47,−69,27	153,0,0
183	RH_Default_Par_2	Parietal	54,−50,28	228,113,122
184	RH_Default_Par_3	Parietal	51,−59,44	234,60,83
185	RH_Default_Temp_1	Temporal	47,13,−30	240,128,128
186	RH_Default_Temp_2	Temporal	61,−13,−21	255,69,0
187	RH_Default_Temp_3	Temporal	55,−6,−10	165,42,42
188	RH_Default_Temp_4	Temporal	63,−27,−6	255,0,0
189	RH_Default_Temp_5	Temporal	52,−31,2	123,17,19
190	RH_Default_PFCv_1	Ventral Prefrontal Cortex	51,28,0	255,64,64
191	RH_Default_PFCd/m_1	Dorsal/Medial Prefrontal Cortex	5,37,−14	220,20,60
192	RH_Default_PFCd/m_2	Dorsal/Medial Prefrontal Cortex	8,42,4	227,66,52
193	RH_Default_PFCd/m_3	Dorsal/Medial Prefrontal Cortex	6,29,15	215,59,62
194	RH_Default_PFCd/m_4	Dorsal/Medial Prefrontal Cortex	8,58,18	203,65,84
195	RH_Default_PFCd/m_5	Dorsal/Medial Prefrontal Cortex	15,46,44	255,83,73
196	RH_Default_PFCd/m_6	Dorsal/Medial Prefrontal Cortex	29,30,42	206,32,41
197	RH_Default_PFCd/m_7	Dorsal/Medial Prefrontal Cortex	23,24,53	232,0,13
198	RH_Default_pCunPCC_1	Precuneus/Posterior Cingulate Cortex	12,−55,15	196,30,58
199	RH_Default_pCunPCC_2	Precuneus/Posterior Cingulate Cortex	7,−49,31	255,64,64
200	RH_Default_pCunPCC_3	Precuneus/Posterior Cingulate Cortex	6,−58,44	211,0,63
